# Mobile human brain imaging using functional ultrasound

**DOI:** 10.1126/sciadv.adu9133

**Published:** 2025-06-18

**Authors:** Sadaf Soloukey, Luuk Verhoef, Frits Mastik, Michael Brown, Geert Springeling, Bastian S. Generowicz, Djaina D. Satoer, Clemens M. F. Dirven, Marion Smits, Borbála Hunyadi, Sebastiaan K. E. Koekkoek, Arnaud J. P. E. Vincent, Chris I. De Zeeuw, Pieter Kruizinga

**Affiliations:** ^1^ Department of Neuroscience, Erasmus MC, Wytemaweg 80, 3015 CN, Rotterdam, Netherlands.; ^2^ Department of Neurosurgery, Erasmus MC, Wytemaweg 80, 3015 CN, Rotterdam, Netherlands.; ^3^ Department of Experimental Medical Instrumentation (EMI), Erasmus MC, Wytemaweg 80, 3015 CN, Rotterdam, Netherlands.; ^4^ Department of Radiology and Nuclear Medicine, Erasmus MC, Wytemaweg 80, 3015 CN, Rotterdam, Netherlands.; ^5^ Signal Processing Systems, Delft University of Technology, Mekelweg 4, 2628 CD, Delft, Netherlands.; ^6^ CCO of BlinkLab Ltd., 216 St. George, Perth, Australia.; ^7^ Netherlands Institute for Neuroscience, Royal Dutch Academy for Arts and Sciences, Meibergdreef 47, 1105 BA, Amsterdam, Netherlands.

## Abstract

Imagine being able to study the human brain in real-world scenarios while the subject displays natural behaviors such as locomotion, social interaction, or spatial navigation. The advent of ultrafast ultrasound imaging brings us closer to this goal with functional ultrasound imaging (fUSi), a mobile neuroimaging technique. Here, we present real-time fUSi monitoring of brain activity during walking in a subject with a clinically approved sonolucent skull implant. Our approach uses personalized 3D-printed fUSi helmets for stability, optical tracking for cross-modal validation with functional magnetic resonance imaging, advanced signal processing to estimate hemodynamic responses, and facial tracking of a lick licking paradigm. These combined efforts allowed us to show consistent fUSi signals over 20 months, even during high motion activities such as walking. These results demonstrate the feasibility of fUSi for monitoring brain activity in real-world contexts, marking an important milestone for fUSi-based insights in clinical and neuroscientific research.

## Teaser

A simple but ultrafast ultrasound probe attached to a custom helmet images live human brain activity during walking.

## INTRODUCTION

Being able to study the human brain in real-world scenarios while the subject is displaying natural behaviors such as locomotion, social interaction, or spatial navigation could be immensely valuable for our clinical and neuroscientific understanding of human brain activity (*1*, *2*). Unfortunately, functional brain imaging techniques come with technical trade-offs, which, until now, have not been able to facilitate high-quality imaging of human brain activity in these ecological contexts. Noninvasive, wearable techniques such as electroencephalography (EEG) (*3*) or functional near-infrared spectroscopy (*4*, *5*) do facilitate mobility but without allowing for high-resolution imaging of deeper brain structures. Optical techniques such as diffuse optical tomography (*5*, *6*) allow for noninvasive imaging with a large field of view (FOV) but have low resolution and limited penetrative depth. Invasive techniques such as electrocorticography (*7*) or intracranial EEG (*8*) allow for high-resolution mapping of the brain but require implantation beneath the skull, with limited implant lifetime. Last, noninvasive, whole-brain techniques such as magnetoencephalography (*9*) and especially functional magnetic resonance imaging (fMRI) (*10*) dominate the current human functional brain imaging landscape but require very large and expensive machinery while severely restricting the movement of subjects during imaging. Many functional paradigms such as locomotion or speech are performed in an “imagined” fashion, where the subject is asked to imagine performing the functional task instead (*11*–*13*).

With the advent of ultrafast Doppler ultrasound imaging, a brain imaging technique called functional ultrasound imaging (fUSi) has emerged for neuroscientific (*14*, *15*) and clinical use (*16*–*18*). fUSi exploits a high frame rate acquisition scheme using unfocused transmissions to boost the sensitivity of conventional Doppler ultrasound (*14*). This gain in sensitivity allows for detection of subtle changes in hemodynamics in the brain’s (micro)vasculature, which, through the principle of neurovascular coupling, serves as a proxy for functional brain activity (*14*, *19*, *20*). What makes fUSi unique is its ability to combine high-resolution (spatial resolution, ~200 μm) and in-depth (FOV, ~5 cm) functional brain imaging with a high level of portability and flexibility (*16*, *18*). Simultaneously, fUSi is contrast agent–free and comes with all the other well-known benefits of conventional ultrasound: It is real-time, noninvasive, easy to use, and comparatively cost-effective. None of the now available brain imaging techniques are able to combine all these beneficial characteristics at once (*15*, *17*).

Given these benefits, fUSi has found its way from a preclinical context to in-human, neurosurgical applications in less than a decade (*16*, *18*, *21*). During awake brain surgeries for tumor resections, fUSi has been applied successfully to map out hemodynamic-based functional brain activity at a mesoscopic scale, while patients performed language, motor, and sensory tasks (*16*, *18*, *22*). So far, the neurosurgical context has not just been an interesting use case for fUSi; it is a necessary one as well. Given the substantial attenuation and aberration of ultrasound signal through human skull bone (*23*), a craniotomy is a necessary acoustic window to the human brain, making fUSi not applicable outside of the operating room as of yet. As a consequence, fUSi is confined to the very limited context of awake brain surgeries, where time is limited and patients are physically restricted (*17*).

Clinical practice and scientific literature demonstrate other approaches to still achieve acoustic access to the brain outside of the operating room. Clinical use of burrholes or cranioplasties also facilitates—as a by-product—artificial acoustic windows to the human brain (*24*). Cranioplasties are performed in clinical contexts for a range of etiologies where a skull bone defect (SBD) cannot be covered with the patient’s autologous skull bone flap, such as after traumatic brain injury or bone flap infection after intracranial tumor surgery (*25*). The SBD can then be covered by an artificial, patient-specific implant made of materials such as titanium mesh, polymethyl methacrylate (PMMA), or polyetheretherketone (PEEK) (*26*). The latter two types of plastic are far more homogeneous and present with much less acoustic signal attenuation as compared to human skull bone (*27*), making them sonoluscent. A handful of studies have demonstrated the potential of PMMA and PEEK for conventional B-mode ultrasound imaging, e.g., in the context of bedside monitoring of ventricular size in patients with neurotrauma (*28*–*32*).

Recently, Rabut *et al*. (*33*) published the first demonstration of in-human fUSi through a custom, thinned-out PMMA window, implanted in a human subject with an SBD after trauma. The authors show the feasibility of fUSi for mapping and decoding of task-modulated cortical activity through PMMA during functional tasks involving gaming and guitar playing (*33*).

Here, we show the next step: successful use of fUSi in a human subject during locomotion. We show how a conventional PEEK cranioplasty provides enough acoustic transmission to perform detailed fUSi of the sensorimotor cortex of the lip, consistently over multiple repetitions and spread out over a period of close to 2 years. We first describe a personalized three-dimensional (3D)–printed fUSi helmets to fixate the ultrasound probe on the subject’s head, to ensure stability of the probe during walking tasks and enable reproducibility of the same 2D imaging plane across measurements.

Next, we set up an optical tracking pipeline of the subject’s face and ultrasound probe, which allowed for computed tomography (CT)/(f)MRI coregistration of our fUSi data, as well as tracking of facial movements during functional tasks. Functional tasks focus on sensorimotor activation of the lips and include lip licking, lip pouting, and sensory stimulation of the lips. An extensive set of measurements, including several task variations and functional controls, is performed over a period of nearly 2 years. Our work shows reproducible and consistent fUSi signal over a period >20 months, with robust acquisitions of functional brain signal even during high motion scenarios such as walking, as shown in this paper. These results demonstrate the feasibility of fUSi for monitoring human brain activity in ecological contexts and serve as an important milestone toward fUSi-based discoveries of the human brain in clinical and neuroscientific context.

## RESULTS

### Subject recruitment

We recruited two male subjects in their 30s with a PEEK cranioplasty (Johnson - Johnson, DePuy Synthes) to participate in our study. Subject #1 received a left-sided hemicraniectomy and PEEK implant after high-velocity trauma, causing multiple cerebral contusions and postoperative neurological deficits. Most pronounced was the subject’s aphasia, which was considered severe based on baseline linguistic assessment (data S1). In addition, although the subject retained the ability for independent walking, he had motor deficits in the right arm and leg, resulting in a right-sided limp. Subject #2 received a PEEK implant over the right-sided frontotemporal region after the surgical removal of a low-grade astrocytoma in the right insular region. At the time of inclusion, the subject had been tumor progression free for multiple years. No cognitive, motor, or language deficits were reported or objectified at baseline.

Experiments were conducted over a period of nearly 2 years. Subject #1 participated in a total of seven measurements during this period. Subject #2 died during the course of the study due to tumor regrowth and participated in a total of two measurement sessions. The results of these measurements will be shown in this manuscript. However, the main portion of the presented data, including the walking experiments, will involve subject #1 only. More details on subject characteristics can be found in data S1.

### fUSi helmet design

**Fig. 1.**
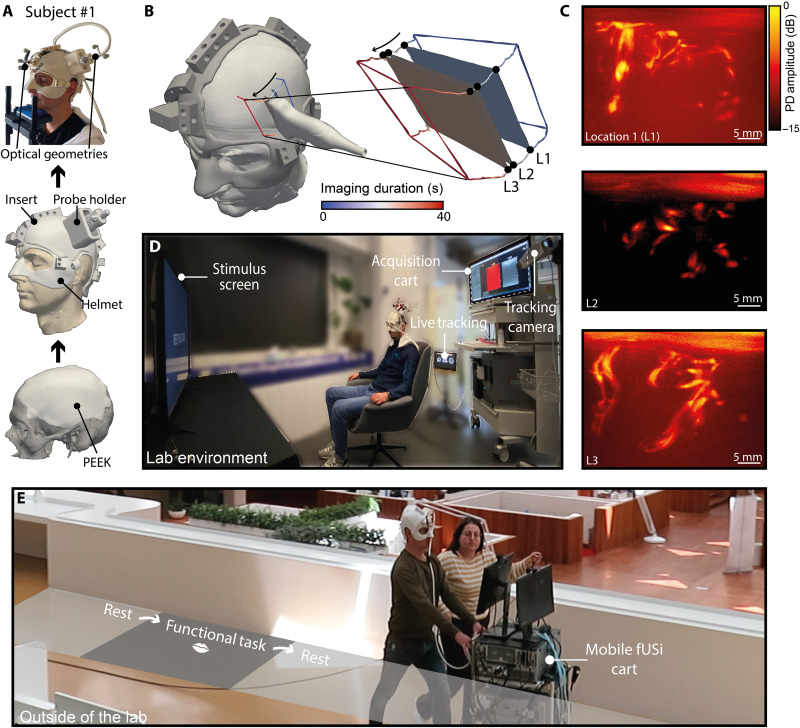
Key components of our experimental setup. (**A**) To facilitate stable and reproducible fUSi throughout the experiment, we designed a custom 3D-printed helmet based on the subject’s MRI. The helmet consisted of a probe insert, which allowed us to revisit the same 2D plane over time, positioned over brain ROIs for functional tasks. Optical geometries were attached to the helmet and probe to monitor their relative position, facilitating fUSi-MRI coregistration and offline 3D reconstruction of the ultrasound path. (**B**) Example of an ultrasound path as reconstructed using our offline optical tracking data. In this case, the probe was moved over the subject’s head using a linear translation (see the directions of arrows). Three interesting imaging locations in terms of vasculature (L1 to L3) were marked within this path, as examples to showcase. (**C**) power Doppler images (PDIs) of interesting brain vasculature seen at three showcase locations (L1 to L3), as marked in (B). A full overview of the vascular images acquired within this path can be found in movie S1. (**D**) Overview of the experimental setup in our laboratory environment. The subject was placed in front of a screen showing the stimulus. The equipment, including our tracking camera, our custom fUSi acquisition cart, and our live tracking tool based on our optical tracking, is also displayed. (**E**) Example of an experimental acquisition using our mobile fUSi cart outside of the laboratory environment. The cart was pushed by the subject himself while performing the task. Permission was required from the subject to publish images.

To facilitate the reproducibility of our measurements over time, we first designed and tested personalized 3D-printed polylactic acid (PLA) helmet (dddrop bv, CAD2M) to fixate the fUSi probe with respect to each subject’s brain anatomy. The helmet was based on the subject’s head contour as extracted from MRI scans and contained two optical geometries necessary for optical tracking (Fig. 1A). To ensure stability of the probe during functional tasks and enable reproducibility of the same 2D imaging plane across measurements, probe inserts were designed on the basis of targeted brain regions of interests (ROIs) for functional tasks (Fig. 1A). More details on the pipeline to design and 3D print personalized helmets and probe inserts can be found in data S2.

### Optical tracking and experimental setup

The position and orientation of the ultrasound probe (GEL9-D, General Electric, USA) and helmet were tracked continuously using an optical tracking camera (Polaris Vega, Northern Digital Inc., Canada) combined with our custom software (data S3). The tracking data were also saved for later use, facilitating offline reconstruction of the ultrasound path, as demonstrated in Fig. 1B and movie S1. These reconstructions allowed us to determine the location of the ultrasound probe relative to the subject’s anatomy, for each of the power Doppler images (PDIs) we acquired during, e.g., a linear translation of the probe over the subject’s head (Fig. 1B).

fUSi acquisitions were first performed in our laboratory environment dedicated to in-human imaging (Fig. 1C). Our aim was to create an ecological environment in which subjects could move within the restraints of being tethered by the cord of the ultrasound probe. Once the signal and helmet proved robust, we moved outside of the laboratory on several occasions using a mobile cart version of our experimental research system for experimental acquisitions during locomotion (Fig. 1D). During our acquisitions, several data streams were acquired and stored synchronously, as can be appreciated in data S4. These data streams included video recordings of the subject’s face, and the movement of which was tracked for functional analyses.

### Validation of anatomical localization of the probe

**Fig. 2.**
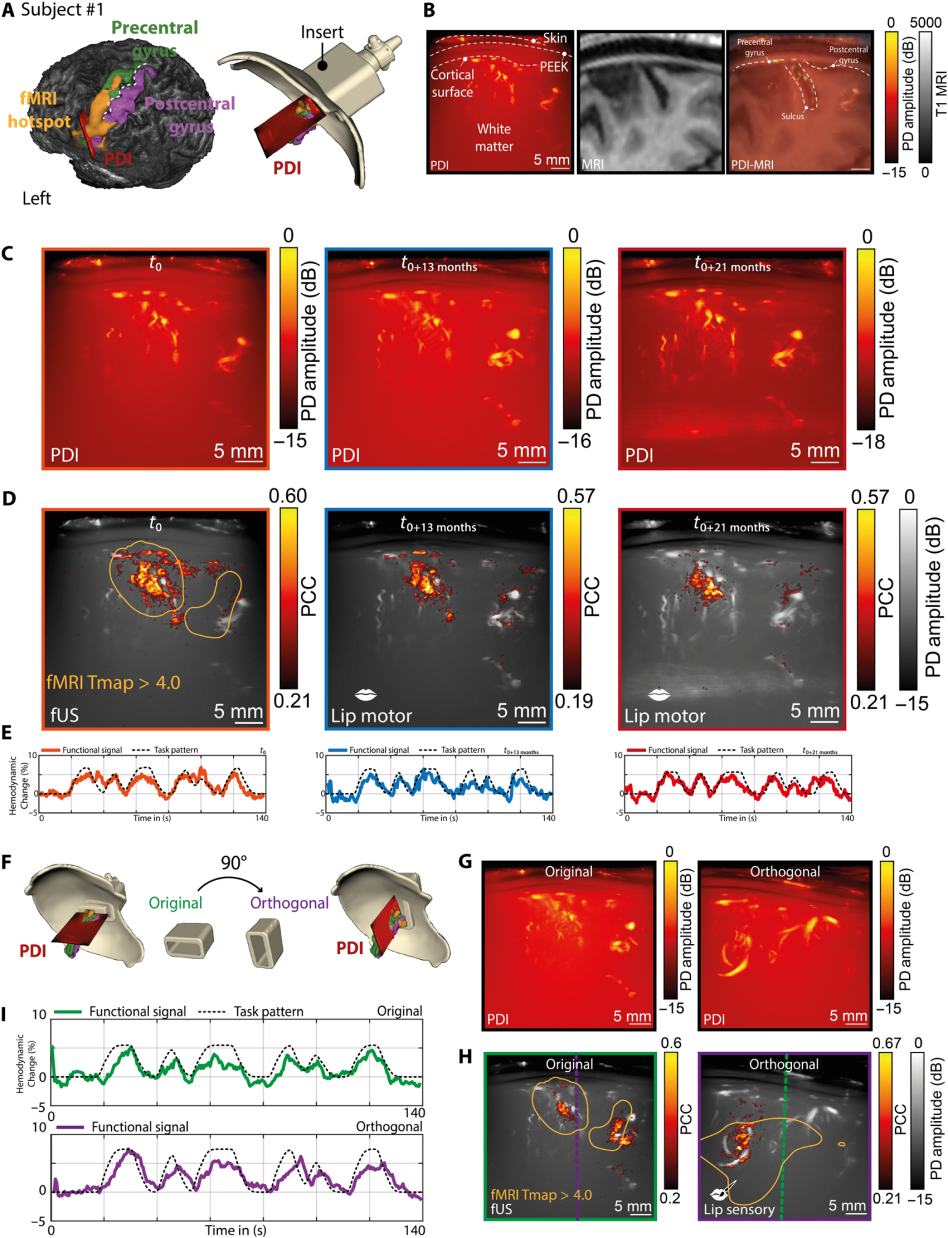
Functional localization and reproducibility of the sensorimotor signal of the lip over time in subject #1. (**A**) The ROI was planned around the sensorimotor cortex of the mouth, overlapping with the fMRI hotspot found during a prior lip pouting task in the same subject. (**B**) The accuracy of the PDI localization could be confirmed by the overlap with the gyri and sulci contour in MRI. (**C**) In subject #1, we were able to revisit the same PDI plane at three time points (*t*_0_, *t*_0+13 months_, and *t*_0+21 months_), demonstrating the reliability of the personalized helmet and insert combination over time. (**D**) At those same three time points, we were also able to consistently map out functional brain activity during a motor lip pouting task, demonstrating the robustness and reproducibility of the fUSi signal. The first panel also demonstrates how the functional region, as found with fUSi, overlapped with the fMRI hotspot, as found during a similar lip pouting task (orange contour). The limits of the color maps shown for each of the time points were based on the minimum and maximum PCC value present in each of the datasets. (**E**) The corresponding functional signals as found at each of the time points depicted in (C) and (D). (**F**) To further study the robustness of the signal, we designed a new orthogonal insert with a probe positioned at a 90° angle relative to the original ROI for a lip sensory task. (**G**) The PDI plane of the original versus the orthogonal insert. (**H**) Functional maps of the original versus the orthogonal insert, with the orange contour showing the relative overlap with the hotspot in fMRI [see also (A)]. The green and purple dotted lines visualize the intersectional plane between the original and orthogonal PDI. (**I**) Functional signals corresponding to the functional maps in (H).

**Fig. 3.**
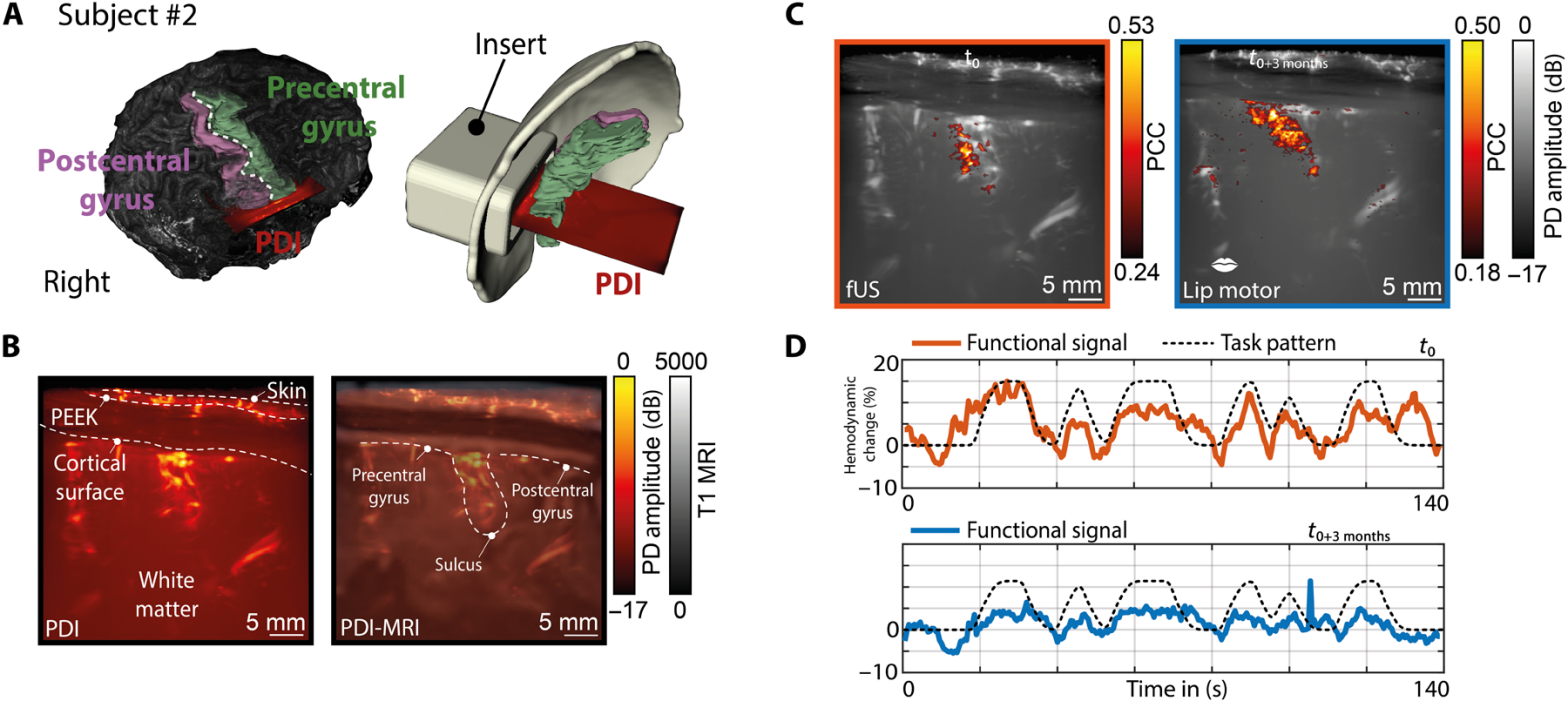
Functional localization and reproducibility of the sensorimotor signal of the lip over time in subject #2. (**A**) The ROI was planned around the sensorimotor cortex of the mouth. (**B**) The accuracy of the PDI localization could be confirmed both anatomically and by the overlap with the gyri and sulci contour in MRI. (**C**) In subject #2, we were able to revisit the same PDI plane at two time points (*t*_0_ and *t*_0+3 months_), demonstrating the reliability of the personalized helmet and insert combination over time. At those same two time points, we were also able to consistently map out functional brain activity during a motor lip pouting task. (**D**) The corresponding functional signals as found at each of the time points depicted in (C) (*t*_0_ and *t*_0+3 months_).

In both subjects, we designed inserts that positioned the probe and fUSi plane directly over the precentral and postcentral gyrus of the left (in subject #1) (Fig. 2A) and right (in subject #2) (Fig. 3A) hemispheres. The precentral and postcentral gyri correspond to the primary motor and sensory cortex, respectively, and care was taken to plan the insert over the lower section of the homunculus (*34*), traditionally involved in sensorimotor control of the mouth, lip, and tongue. Literature contains multiple examples of studies demonstrating the somatotopy of sensorimotor activation of the lips (*34*, *35*), albeit no strong evidence exists for somatotopy of the upper versus lower lip or the right versus left side of the lip, with often heterogeneous bihemispheric activation (*36*–*42*). This sensorimotor area was of particular interest, as the anatomical localization of the PEEK in both subjects allowed for easy access. In addition, we wished to perform a similar task in fUSi as in fMRI for validation purposes, for which the sensorimotor cortex of the mouth seemed particularly useful. Last, our previous fUSi work in the context of awake neurosurgical procedures involved many iterations of lip pouting or lip licking tasks, which ensured our familiarity with the functional paradigm in the context of fUSi (*18*, *22*). The correct positioning of the fUSi probe was verified in real time with our optical tracking system (data S4). Coregistration of the PDIs and corresponding MRI slice confirmed correct localization based on vascular patterns following gyri and sulci (Figs. 2B and 3B).

The acoustic properties of PEEK are distinctly different from that of brain tissue, leading to the distortion of our ultrasound signal through the PEEK material. In data S5, we study the nature of these distortions and provide a quick fix and an exact algorithmic solution to obtain undistorted ultrasound images through PEEK, so that they can be matched with other modalities such as, in our case, with fMRI. For the rest of the study, where we only study the fUSi data without reference to other modalities, we used the quick fix solution, which entails image reconstruction using a higher global sound speed to account for the increased sound speed in PEEK with respect to brain tissue. This quick fix solution allows for similar resolution and vascular details to be observed as with the aberration correction method (for comparison, see data S5) yet at a much faster computation time, facilitating real-time feedback during the experiments and fast postprocessing of the results.

### Validation of functional localization of the probe using fMRI

Functional mapping using fUSi is accomplished by acquiring a series of sequential PDIs to measure changes in cerebral hemodynamics. The spatiotemporal changes between these images serve as a proxy for neural activity through the process of neurovascular coupling (*14*, *19*, *20*). In the recently presented experiments, PDIs were acquired with an imaging frame rate of 4 Hz.

We wanted to first confirm the presence of functional activity within our fUSi image before expanding our experiments. Therefore, we performed a simple ON-OFF lip pouting (motor) and lip brushing (sensory) task (Figs. 2, C to E, and 3, C and D) in both subjects, to create fUSi maps depicting functional pixels [defined as pixels that showed a Pearson correlation coefficient (PCC) value of >3× the SD of the mean PCC value of voxels in a predefined noise region]. To perform our PCC analyses, the stimulus pattern was first convolved with an estimated hemodynamic response function (HRF) (*43*–*47*). As far as we know, there are no data available on the human HRF for fUSi. We therefore estimated an HRF ourselves using four training datasets in which the subject performed a lip licking task while walking. This training set was obtained 1 week before the experiments shown here. The HRF was found then by minimizing the error between the measured fUSi signal and the task time course convolved with the HRF kernel. The HRF kernel itself was modeled as a weighted sum of basis functions. Specific details of this procedure can be found in data S6.

We were able to demonstrate overlap in fUSi-based functional regions as compared to coregistered fMRI hotspots, as found during a prior lip pouting task in fMRI (Fig. 2, A and D). Previous work by our team showed similar consistent overlap between fUSi and fMRI regions in subjects imaged during awake neurosurgical procedures (*22*). More details on the functional tasks used for fUSi and fMRI can be found in data S7.

Given the limited FOV of our single 2D plane, we chose to also confirm the robustness of the functional hotspot by designing a new insert for subject #1, with the probe positioned at a 90° angle relative to the original ROI, using our optical tracking data. The orthogonally positioned PDI intersected the fMRI hotspot as well and again confirmed the presence of a functional region during a sensory lip brushing task (Fig. 2, F and G).

### Reproducibility and consistency of the fUSi signal over time

In both subject #1 and subject #2, we could reproducibly map functional brain activity during both sensory (lip brushing) and motor (lip pouting) tasks over a period up until 21 months (Figs. 2, C to E, and 3, C and D). This demonstrates how the fUSi signal could be acquired reproducibly and consistently over longer periods of time using our helmet and insert combination. For subject #1, a slight improvement in vascular detail was seen at *t*_0+21 months_ (Fig. 2C), which was due to improved acoustic contact between the ultrasound probe and the subject’s skin. In all measurements, we made use of ultrasound gel (AquaSonic) to improve acoustic coupling; however, factors such as length of hair slightly influenced the quality of the acoustic contact over time (see also Materials and Methods).

### Functional specificity of the fUSi signal

The functional region delineated during the motor task (lip pouting; Fig. 2, B to E) localizes primarily in the precentral gyrus, as to be expected. However, the sensory task performed in subject #1 (Fig. 2, G and H, left) localizes in both the precentral and postcentral gyri, indicating potential involvement of both sensory and motor cortices. Although this may raise the question to what extent the hemodynamic-based fUSi mapping is spatially selective, it should be noted that the primary sensory prominently projects to motor cortex, often eliciting coactivation upon electrical stimulation (*48*–*51*).

**Fig. 4.**
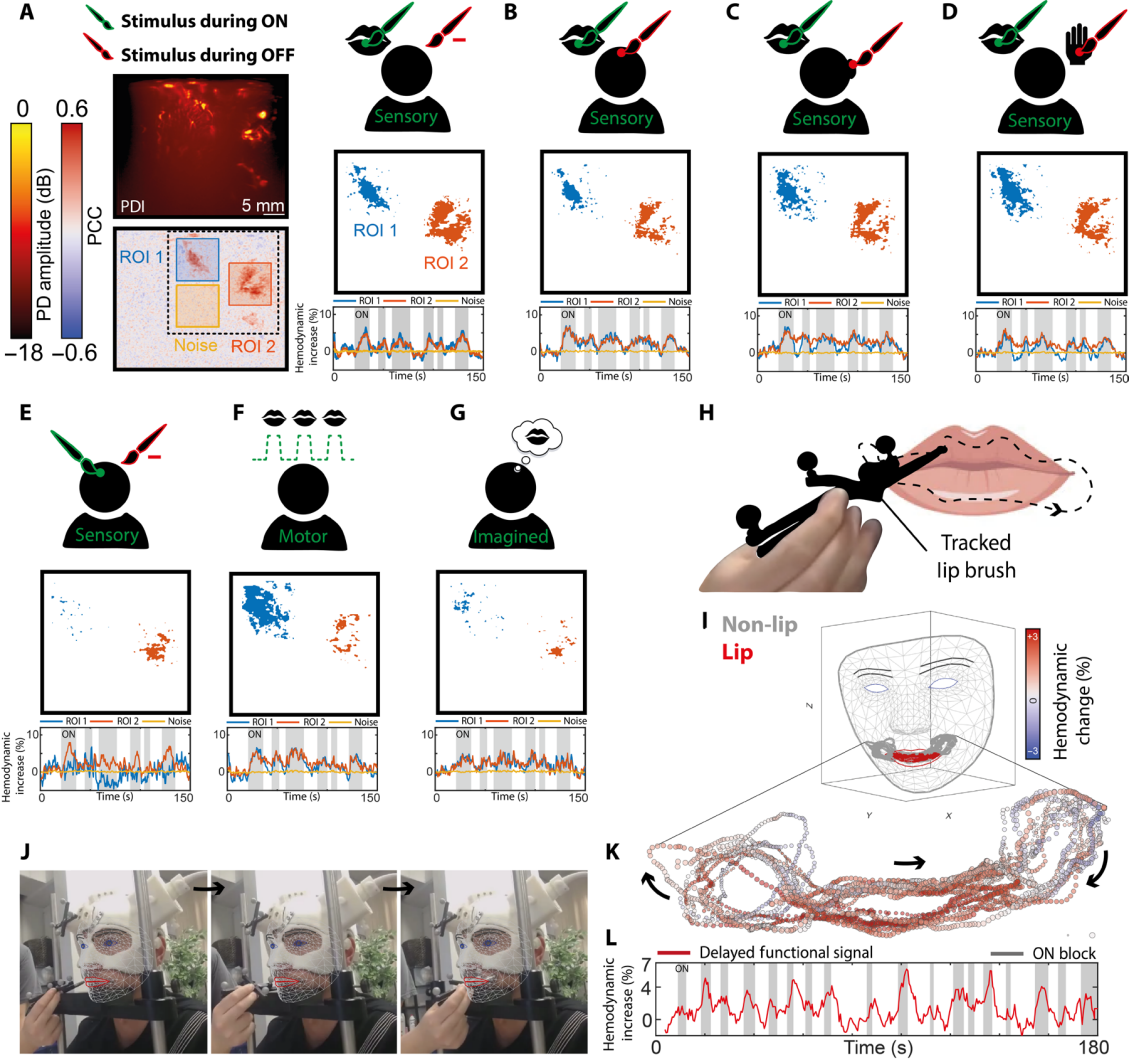
Demonstrating the functional specificity of the fUSi signal in subject #1. (**A**) A series of task variations in the lip sensory task. An ON (lip brushing)–OFF (rest) sensory lip task evokes a reproducible functional fUSi map with two distinct functional regions [ROI 1 (precental gyrus) and ROI 2 (postcentral gyrus)]. The adjacent noise region used for functional thresholding is marked in yellow. The thresholded functional map (PCC > 3× SD of noise signal) highlights two distinct functional regions. (**B**) Alternating ON condition (lip brushing) with the OFF task of brushing the forehead produces a similar functional map. (**C**) Alternating ON condition (lip brushing) with the OFF task of brushing the right ear produces a similar functional map. (**D**) Alternating ON condition (lip brushing) with the OFF task of brushing the hand produces a similar functional map. (**E**) Brushing the forehead only (ON), with a rest condition in OFF times, leads to far less functional activation, only present in ROI 2. (**F**) A motor task (lip pouting) does give functional activity but different in the spatial pattern, with more prominent localization in the precentral gyrus (ROI 1). (**G**) The imagined version of the task shown in (A) resulted in a similar functional map as seen in (A) to (D), although less prominent. (**H**) Next, we performed a continuous lip brushing task, using optical tracking to determine the brush position. (**I**) Using the MediaPipe library by Google (*52*), we projected the lip brushing trace over a mesh representation of the subject’s facial anatomy. (**J**) Three example time points with different lip brushing positions. (**K**) Plotting the average percentual hemodynamic change in our ROIs as a function of brush location demonstrates a specific rise in the hemodynamic signal only when the lip itself was brushed. (**L**) Delayed functional signal found in the ROI when the brush was in the lip region (defined as “ON blocks”).

To further study the specificity of the functional signal using fUSi, we introduced several task variations in subject #1, as can be seen in Fig. 4. A classic ON (lip brushing)–OFF (rest) sensory lip task evoked a reproducible functional fUSi map with two distinct functional regions (ROIs 1 and 2; Fig. 4A), as was also seen in Fig. 2. Alternating the ON task with a different OFF task such as brushing the forehead (Fig. 4B), the right ear (Fig. 4C), or the hand (Fig. 4D) did not notably alter the functional map.

As a control, we performed a forehead-brushing task only, with no involvement of the lip (Fig. 4E), where we see close to no activation, with only a small number of functional pixels in ROI 2. These findings suggest that the region of the sensorimotor cortex we are imaging seems lip specific, not involving the functional activity of adjacent regions on the sensorimotor homunculus.

A motor task (lip pouting) in the same task pattern, as shown in Fig. 3A, did give functional activity in the same region of our ROIs but different in the spatial pattern (Fig. 3F), with more involvement of the precentral gyrus, as is to be expected. Asking the subject to imagine lip pouting in the ON times, similar to the imagined task variations performed in fMRI studies (Fig. 3G) (*11*–*13*), resulted in a similar functional map, as seen in Fig. 3 (A to D), although less prominent. Details on the exact specification of these functional tasks can be found in data S7.

### Mapping functional signal in a continuous task

Once the specificity of the functional signal was confirmed, we set out to perform a functional task without a predictable ON-OFF pattern, to further study the robustness of the functional signal. The task consisted of continuous lip brushing by one of the researchers (Fig. 4H). The tracked brush was moved continuously for several minutes in an unpredictable pattern over the upper and lower lip, as well as adjacent parts of the face. Using the optical tracking of the helmet, as well as the brush itself, we could determine the position of the brush relative to the subject’s facial anatomy accurately at each time point of the experiment. Using the MediaPipe library by Google (*52*), we could then extract the facial parameters such as the FaceMesh, Blendshape coefficients, and Rotationmatrix from a video file of the face taken during the experiments (more explanation on this in Materials and Methods). Hence, we could project the brush trace over a mesh of the subject’s face, allowing us to discern time points where the brush touched the lip versus the adjacent cheeks (Fig. 4I). Plotting the average percentual hemodynamic change in our ROIs as a function of brush location demonstrates a specific rise in hemodynamic signal only when the lip itself was brushed versus the surrounding face (Fig. 4, J and K), again conforming the specificity of the signal.

### fUSi using a mobile cart to facilitate walking

After studying the fUSi signal extensively in sedentary position using the helmet, we proceeded to an experimental setup during walking. The ultrasound acquisition system could be pushed by subject #1, while he was still tethered to the acquisition system with the ultrasound probe (Fig. 1E). The cart contained two separate screens to display the functional task video and the real-time PDIs during acquisition. A long (100 m) extension cord was used to allow for a large range of motion. Further details on the acquisition system can be found in Materials and Methods.

**Fig. 5.**
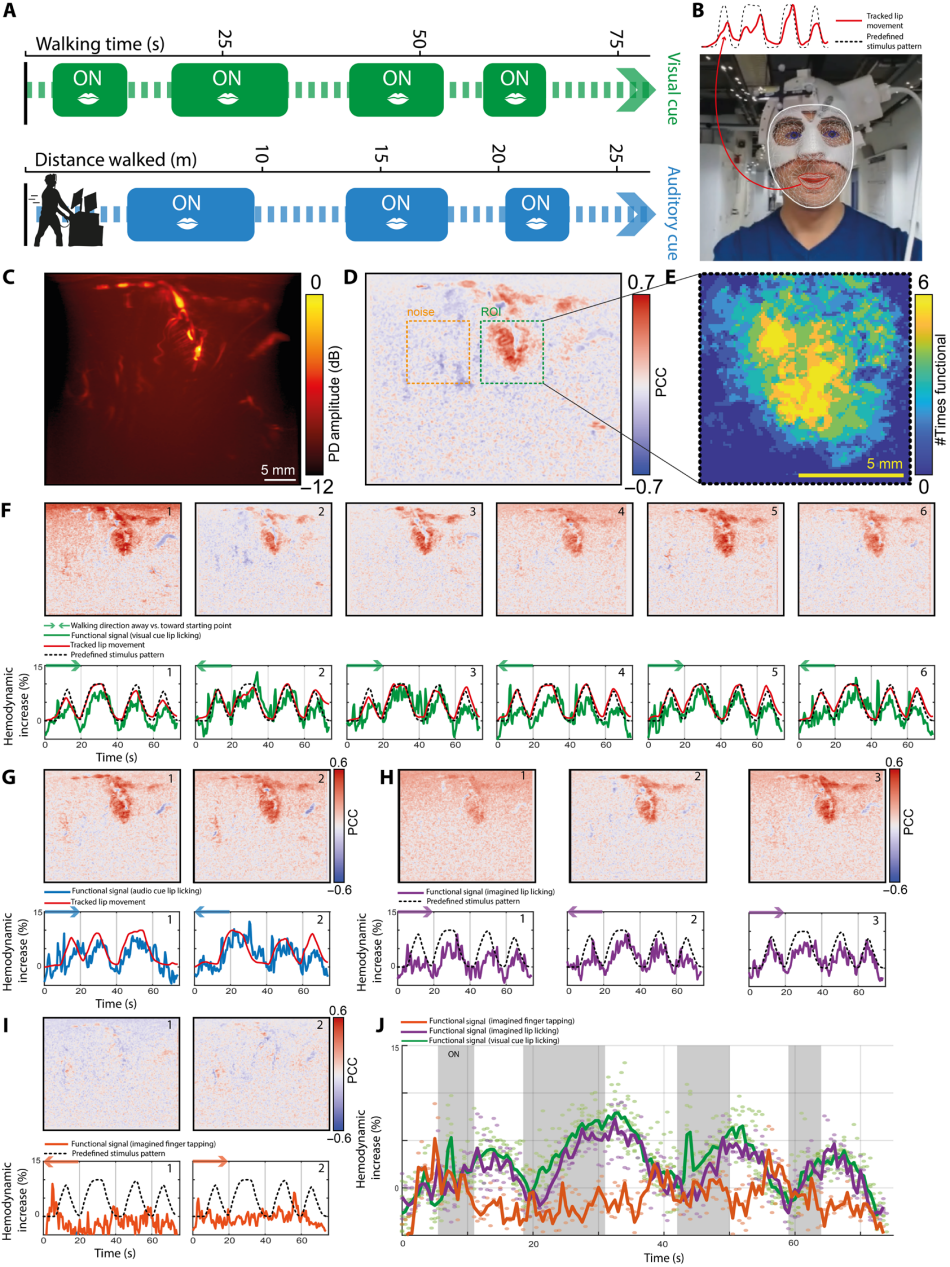
fUSi signal acquired during walking. (**A**) Overview of experimental conditions, where ON blocks (lip licking, finger tapping, or imagined lip licking) were either introduced with a visual cue (video on screen) or audio cue (ON-OFF indicated verbally based on landmarks on the ground). (**B**) Example video footage taken from the subject during walking and used to analyze facial movements using the MediaPipe library by Google (*52*). The red trace represents an example of the tracked lip movements. (**C**) Example of the averaged PDI acquired during walking. (**D**) Example of a fUSi map acquired during a visual lip licking task [see (A)], with the ROI marked in green. The adjacent noise region used for functional thresholding is marked in yellow. (**E**) A total of six iterations of the task shown in (D) were performed. Here, we summarize the presence of functional pixels (>3× SD of the noise signal) in the ROI across six iterations. Pixels with a higher summarized value were present in more iterations of the task, highlighting the robustness of their functional involvement. (**F**) Six example iterations of the same functional task as shown in (D) (visual-based lip licking), showing the reproducibility and robustness of the functional signal captured during locomotion. (**G**) Example traces of auditory-based lip licking task. (**H**) Functional maps and signal in three iterations of the imagined lip licking task show similar activation as in (E), although slightly less pronounced in signal amplitude. (**I**) As a control condition, imagined finger tapping did not evoke any functional signal. (**J**) Summarizing scattering plot showing the functional signal in the lip licking task (green), imagined lip licking task (purple), and imagined finger tapping task (orange).

During recordings of a little over 1 min each, the subject (Fig. 5A) was asked to walk a continuous straight line of a maximum of 30 m while pushing the mobile fUSi cart and following a functional task video displayed on the screen. The subject would walk this continuous straight line in both directions, both away and toward the starting point, to avoid unnecessary delays in successive recording iterations. A camera recorded the subject’s face continuously to allow for post hoc functional analyses of facial and especially lip movement (Fig. 5B). The subject was first asked to perform a simple ON-OFF lip licking task while walking, both based on a visual ON-OFF cue in video form and an audio ON-OFF cue, given verbally by the experimenter based on certain landmarks on the ground (Fig. 5A). More details on the functional tasks used can be found in data S7 and movie S2.

### Reproducible and consistent fUSi signal during walking

We were able to produce high-quality PDIs (Fig. 5C) and functional maps (Fig. 5, D and E) while acquiring a lip licking functional signal during locomotion. These functional maps were consistent and reproducible over multiple iterations, both when using a visual cue (Fig. 5F) and an audio cue (Fig. 5G). Close to no motion correction was necessary during the preprocessing of our PDI stacks, indicating a high level of in-plane stability during image acquisitions (data S8). A similar signal could be acquired when asking the subject to imagine lip licking while looking at the functional video, without actively performing the task (Fig. 5H). A control task with imagined finger tapping did not result in any functional activation, confirming again the specificity to the imagined lip pouting signal (Fig. 5I). A summarizing scatterplot in Fig. 5J demonstrates the specificity of the lip-related functional signal acquired during walking. Movie S2 shows an example functional recording of the lip licking task, as shown in Fig. 5F.

## DISCUSSION

This paper demonstrates robust and reproducible fUSi of human brain activity during walking. Using a conventional ultrasound probe and a 3D-printed personalized helmet, we were able to consistently revisit the same ROIs in two subjects, despite the challenges that come with repeated 2D imaging. We were able to demonstrate activation of the sensorimotor cortex of the mouth with both motor and sensory tasks, confirmed by colocalization with the respective fMRI hotspot. The functional maps and underlying functional signals were reproducible over a period of up to 21 months. We performed multiple variations of the sensory lip task, confirming the functional region’s lip-specific responsiveness. We were also able to acquire robust and reproducible functional data outside of the controlled laboratory environment, with our subjects walking during our acquisitions, without facing motion-related problems.

Compared to the neurosurgical setting our team is accustomed to, having access to tethered but freely moving subjects who can be imaged over multiple sessions was a game-changer. Many of the control or repetition measurements we show in this manuscript are simply not possible in the intraoperative context given the limited time and constraints on the stamina of the awake subject. However, in light of technology development and validation, repetition is key. Each of our functional measurements produced consistent results, even with months between measurement sessions, demonstrating the robustness of our results.

One notable observation during our experiments is the apparent specificity of the lip-related activation of the sensorimotor cortex, as imaged by fUSi, both in the active and the imagined conditions. Although literature tells us that we could rightfully expect our hemodynamic technique to spatially discriminate representation sites that are in close proximity in the sensorimotor homunculus (*37*, *53*), our first fUSi observations inspire further research. For example, it would be interesting to further unravel the imagined functional task conditions shown in this paper. Although fMRI literature relies heavily on motor imagery paradigms to study sensorimotor brain activity (*11*–*13*, *54*), it is still notable how similar our imagined maps are to the actual motor paradigm, especially in Fig. 5 (F and H). One explanation could be our subject’s misinterpretation of the task: Despite us seeing no detectable movement of the tongue or lip on video recordings during the imagined conditions, the subject could have performed a similar version of the task within the oral cavity and the mouth closed.

Similarly, the consistent coactivation of what would be precentral gyrus during a sensory task, as shown in Figs. 2 and 3, and the persisting functional region found when performing sensory stimulation of the forehead only, as shown in Fig. 4E, warrant further investigation. Similar to the very first homunculus mapped out by Penfield and Boldrey (*34*) in the 1930s using electrical stimulation, it would be interesting to pinpoint how much spatial selectivity we can actually achieve when imaging human cortex using fUSi, a hemodynamic-based signal. These observations can be incredibly informative in light of future ambitions such as fUSi-guided decoding of brain signals for brain-computer interfaces. The first examples in nonhuman primates using fUSi-based decoding of motor intention seem promising (*55*, *56*).

Before any of the above can actually be accomplished, we need to move away from our 2D context, which is spatially limited and sensitive to out-of-plane movements, toward the use of real-time 3D probes. To overcome the challenges of 2D imaging in this study, we used our custom 3D-printed helmet (i) as a reference for the relative probe position to the subject’s neuroanatomy, (ii) as a frame to physically stabilize the probe over an ROI during the task, and (iii) as a means to be able to revisit the exact same 2D plane over multiple imaging sessions. Although the current helmet facilitated all three functions, a 3D fUSi capable probe is likely to further ameliorate artifact correction due to motion, out of plane movements, and/or inconsistencies over multiple imaging sessions. In addition, 3D fUSi will allow us to perform more dynamic mobile mapping of the human brain, opening up possibilities such as dynamic decoding of the sensorimotor cortex during locomotion, which could be extremely valuable in monitoring and active guidance of functional plasticity during rehabilitation programs in patients with tumor or neurotrauma. For this application, however, overcoming the challenge of real-time processing of vast amounts of 3D fUSi data will be particularly essential.

Ultimately, our ambition would be to image not just through cranioplasties such as PEEK but transcranially with the same or even better imaging quality, as demonstrated in this paper. The current inability to do so is one of the major limitations of ultrasound-based techniques in humans. Although literature shows how this limitation may be overcome by using contrast agents and performing so-called transcranial ultrasound localization microscopy (ULM) (*57*, *58*), ULM in functional settings is still unfavorable given the need for intravenous microbubble delivery and long time windows for data acquisitions.

Nevertheless, our work confirms the compatibility of unmodified clinical-grade cranioplastes with fUSi experiments. Part of the appeal of PEEK in particular, in addition to its sonoluscency, is its already widespread clinical use, which could warrant cranioplasties as a new clinical standard of care for, e.g., postoperative monitoring purposes. In the setting of traumatic brain injury, for example, continuous acoustic access to the brain could add an additional imaging modality to monitor functional connectivity in comatose patients in the intensive care unit (ICU), aiding in the prediction of functional outcome and treatment decisions accordingly. Now, only expensive and logistically challenging fMRI scans or EEG measurements are available in the ICU for these purposes (*59*, *60*).

Apart from just for functional imaging, the structural images we could make through PEEK using a commercially available ultrasound machine (see data S9) could open up a new way of postoperative monitoring of tumor (re)growth, without the need to rely on periodical expensive MRI scans, an avenue which so far seems to have remained mostly undiscovered in the clinical context (*29*).

Our current paper follows shortly after the study of Rabut *et al.* (*33*) published in *Science Translational Medicine* (*33*), which was developed in the same time frame. In their paper titled “Functional ultrasound imaging of human brain activity through an acoustically transparent cranial window,” the authors demonstrate their development of a thinned, experimental PMMA cranioplasty implanted in a subject with a hemicraniectomy after trauma. The authors show their ability to capture functional activity during a gaming and guitar strumming task. What sets our study apart is the fact that we demonstrate in-human mobile fUSi: We can capture functional brain activity in a walking human. Furthermore, the longer time span of our measurements has allowed for an in-depth analysis of the functional validity of the fUSi signal and includes many repetitions of datasets and functional task verifications to ensure the robustness, reliability, and reproducibility of the signal. The different task variations we designed throughout the years allow us to confidently couple our functional signal to meaningful brain activity relating to sensorimotor control of the mouth. The swift follow-up of the study of Rabut *et al.* (*33*) and our paper does send out one clear, unified message: Using fUSi through already available, clinical-grade cranioplasties provides unique access to human brain functionality.

Here, we demonstrate that fUSi allows us to map human brain functionality during walking in subjects with sonolucent PEEK cranioplasties, at depths of multiple centimeters, in a robust and reproducible fashion. These observations further fuel the field to consider fUSi as more than just an intraoperative tool, pushing toward the development of mobile, in-human fUSi as a new means to unraveling the human brain in clinical and neuroscientific context.

## MATERIALS AND METHODS

### Subject recruitment

Two subjects with a PEEK cranioplasty were recruited from the Department of Neurosurgery of the Erasmus MC in Rotterdam. Before inclusion, written informed consent was obtained in line with the National Medical Ethical Regulations (MEC-2019-0689 and MEC-2022-0087, NL80307.078.22).

### Baseline linguistic and cognitive assessment

To determine baseline linguistic and cognitive abilities of both subjects, a standard clinical test battery including language tests such as the DIMA and DuLIP (Diagnostic Instrument for Mild Aphasia and Dutch Linguistic Intraoperative Protocol) and cognitive tests such as the Trail Making test was performed by trained clinical linguist (D.D.S.) (data S1).

### 3D-printed personalized helmet

We designed a personalized 3D-printed PLA helmet to fixate the fUSi probe with respect to the subject’s brain anatomy. The helmet was based on the subject’s head contour as extracted from MRI scans and contained two optical geometries necessary for optical tracking (Northern Digital Inc., Canada). A step-by-step explanation of the production pipeline can be found in data S2.

### PEEK cranioplasty

The PEEK cranioplasty of subject #1 was custom designed to cover the left-sided hemicraniectomy and was modeled by the manufacturer (Johnson - Johnson, DePuy Synthes) after the subject’s contralateral, nonaffected skull based on CT scans. Dimensions were 130 mm by 162 mm by 44 mm, with a consistent implant thickness of 4 mm. More details on the implant can be found in data S1.

### Optical tracking and ultrasound path reconstruction

Position and orientation of the fUSi probe and helmet were tracked continuously using an Northern Digital Incorporated (NDI) Polaris Vega optical tracking system (SN P9-04539, Northern Digital Inc., Canada), which was configured to track infrared reflective reference geometries attached to the fUSi probe and helmet. Custom software was designed to record the tracking information featuring six degrees of freedom at an average rate of 20 Hz.

To be able to visualize the fUSi probe’s position relative to the subject’s brain in real time during our experiments, we used the tracking information in conjunction with custom-built software using the Visualization Toolkit (*61*). The tracking data were also saved for later use, facilitating offline 3D reconstruction of the ultrasound path, as demonstrated in Fig. 1B. More details on our optical tracking pipeline can be found in data S3.

### Experimental context

fUSi acquisitions were mostly performed in our laboratory environment dedicated to in-human imaging (Fig. 1C). Our aim was to create an ecological environment in which subjects could freely move within the restraints of being tethered through the cord of the ultrasound probe. On several occasions, we moved our mobile experimental research system to an external location (the hallway of our laboratory building) for experimental acquisitions during locomotion (Fig. 1D). During our acquisitions, several data streams (ultrasound, optical tracking, and video) were acquired and stored synchronously, as can be appreciated in data S4.

### fUSi acquisitions

fUSi acquisitions were performed using an experimental research system (Vantage-256, Verasonics) interfaced with a 9L-D linear array transducer (GE Healthcare; 5.3 MHz). For all scans, we acquired continuous angled plane wave acquisition (10 equally spaced between −12° and 12°) with a pulse repetition frequency (PRF) of 800 Hz. The average ensemble size (number of frames used to compute one PDI) was set at 200 frames from which the live PDIs were computed, providing a live Doppler frame rate (FR) ranging of 4 Hz. The PDIs and the raw, angle-compounded beam-formed frames were stored to a fast PCIe SSD hard disk for offline processing purposes.

### fUSi mobile cart

To facilitate measurements during walking, we loaded the Vantage-256 and analysis PC onto a small cart, which could be pushed by the subject himself. Electrical power was provided through a long 100-m-long extension cord and a safety isolating transformer. The cart contained two screens, one facing the subject and showing the functional video, and one facing the experimental team showing real-time PDIs during acquisition. The mobile cart was equipped with several video cameras, which were captured synchronously with the PDI data (see data S4) using OBS Studio screen recorder (OBS Studio Contributors).

### (f)MRI acquisitions

Each subject underwent a single fMRI scan of maximum 60 min before the fUS acquisitions. MRI was performed at 3.0T with an eight-channel head coil (Discovery MR750, GE Healthcare, Milwaukee, WI, US). Whole brain functional MR images were obtained with a single shot T2* weighted echo planar imaging (EPI) sequence sensitive to blood oxygenation level–dependent contrast with the following parameters: repetition time (TR) = 3000 ms, echo time (TE) = 30 ms, flip angle = 90°, acquisition matrix = 96 × 64, FOV = 240 mm by 180 mm. We acquired 54 slices with a slice thickness of 2.2- and 0.3-mm gap. In addition, one higher temporal resolution scan was performed with TR = 1500 ms and 27 slices covering the brain parenchyma underlying the SBD. All functional data acquisition started with five dummy scans, which were discarded from further analysis. Subjects received instructions and practiced the fMRI task together with a researcher before MRI scanning. During scanning, stimuli were visually presented outside the scanner onto an MRI-compatible monitor that was visible with a mirror mounted on the head coil. In addition, a high-resolution 3D T1-weighted inversion recovery fast spin gradient recalled echo (IR FSPGR) structural MRI was acquired in the axial plane with the following parameters: TR = 7.93 ms, TE = 3.07 ms, inversion time = 450 ms, flip angle = 12°, acquisition matrix = 240 × 240, FOV = 240 mm by 240 mm. One hundred seventy-six contiguous slices were acquired with a slice thickness of 1 mm.

### Functional paradigms

On the basis of the anatomical localization of the PEEK in both subjects, as well as the wish to perform a similar task in fMRI for validation purposes, we chose to focus on the sensorimotor cortex of the mouth using motor (lip pouting) and sensory (lip brushing) tasks. For the motor lip pouting task, the subject was asked to follow a task video in which lip pouting was demonstrated during the ON blocks. For the sensory task, one of the researchers (S.S.) stimulated the subject’s lips using an optically tracked, fine-haired brush during ON times. In the majority of the sedentary, fUSi-based functional tasks displayed in this manuscript, a 140-s task pattern was used with randomized ON-OFF blocks ranging between 4.1 and 15.4 s. For the fMRI acquisitions, longer ON-OFF blocks of 30 s each were used, in parallel with our in-house clinical fMRI acquisition schemes. The functional task used during walking focused on lip licking specifically and involved two task variations, with a total task duration of 74 s each. Detailed descriptions of the functional task patterns used during the fUSi and fMRI acquisitions can be found in data S7.

### fMRI data processing

fMRI analysis was performed offline using Statistical Parametric Mapping (SPM8, Functional Imaging Laboratory, UCL, UK) implemented in MATLAB (vR2015b). For each subject, we first spatially realigned all fMRI images and coregistered these images to the individual’s T1-weighted image, using a rigid body transformation as implemented within SPM8. Functional images were smoothed with a 3D Gaussian full width at half maximum filter of 6 mm by 6 mm by 6 mm. All fMRI data were analyzed using the general linear model, by modeling the experimental and the control conditions in a blocked design (see data S7 for the exact task patterns). The blocks were convolved with the HRF, corrected for temporal autocorrelation, and filtered with a high-pass filter of 128-s cutoff. Motion parameters were included in the model as regressors of no interest to reduce potential confounding effects of motion. Individual t-contrast images for the experimental versus control condition were generated and thresholded individually at ~60% of the maximum *t* value. Resulting thresholded images were projected on the 3D T1-weighted image and visually checked by a neuroradiologist with >20 years’ experience (M.S.) with fMRI to assess that expected activation patterns were detected, with threshold adaptation if necessary.

### Estimating the fUSi HRF

We estimated a fUSi-specific HRF on the basis of four training datasets in which the subject performed a lip licking task while walking, similar to what is shown in Fig. 5. This training set was obtained 1 week prior that of the set used for Fig. 5. The HRF was found the by minimizing the error between the measured fUSi signal and the task time course convolved with the HRF kernel. The HRF kernel itself was modeled as a weighted sum of basis functions. Specific details of this procedure can be found in data S6.

### Extracting lip movements from video using Blendshape

All video streams were synchronously recorded at 60 frames per second using the open-source Broadcast Software named OBS Studio (https://obsproject.com/). In the postprocessing stage, we separated the individual streams again into various subvideos (face, cue, etc.) using a MATLAB (vR2020b) script, with Fast Forward moving picture experts group (FFmpeg) for lossless splitting. We then used the MediaPipe library by Google (*52*) to extract the facial parameters such as the FaceMesh, Blendshape coefficients, and Rotationmatrix from a video file of the face. These parameters were obtained using a Python program and were saved in an HDF5 file to facilitate further processing in conjunction with the overall fUSi processing in MATLAB (vR2020b). We used the “mouthSmileRight” and/or “mouthSmileLeft” Blendshape coefficient for the generation of the stimulus signal. Before correlation, we first used a moving median filter of 0.5 s (31 frames) to remove outliers and fill in missing data points after which we convolved the output with our estimated HRF. Careful inspection between the video and the Blendshape coefficient revealed a 0.4 delay of the coefficient lagging the video. We accounted for this delay in the further processing and plotting.

### Ultrasound distortion correction

We are able to visualize functional activity through the PEEK cranioplasty; however, it is necessary to increase the bulk sound speed used in the delay-and-sum reconstruction to compensate for the higher sound speed of the implant layer. This results in a warping of the reconstructed vasculature and can generate errors when coregistering to structural MRI. We tested whether it is possible to correct this warping by segmenting the skull implant from the reconstructed b-mode images and using a simple multilayer ray-tracing model to update the delays for each voxel (*62*). We found that this approach resulted in better alignment to MRI, as demonstrated in data S5.

### fUSi data processing

All the PDIs and associated results are obtained using postprocessing of the continuous ultrafast ultrasound data that were recorded to a disk during the experiments. Although the variety in experiments and datasets would benefit from tailored processing, we chose, for the sake of clarity, to have the same processing pipeline and parameters for all datasets shown, except for Fig. 4K where additional conditioning of the signal was necessary to obtain the desired result. Every PDI was computed using an ensemble of 800 ultrafast ultrasound frames, with a half overlap between consecutive frames yielding a frame rate of 2 Hz. This relatively large ensemble size provided a very stable functional signal and seems appropriate because of the relative slow hemodynamic response that we have observed (see data S6). A half overlap regains a bit of the loss in temporal resolution. The Doppler signal was obtained using an SVD rank reduction technique that works by removing the first dominant singular vectors which span the stationary tissue signal, from the ensemble of frames, leaving only the blood signal. For all sets, we removed the first 56 vectors (7%) and the last 40 vectors (5%) to reduce noise. Similar to other fUSi publications, including our own, these specific thresholds are not absolute nor are they essential to the end results. We found that for some datasets, a higher clutter cutoff of 10% would provide better functional datasets with higher correlation values, yet for others, this was not the case. For the sake of simplicity, we therefore chose one high and one low cutoff value for all datasets. After this Doppler filtering step, we discarded 40 Doppler frames with the highest median absolute deviation (MAD) per frame. This step ensures that possible outlier frames are discarded in the subsequent averaging step. We found that this step provided a slightly smoother pixel time course which benefits the functional analysis after. The remaining 760 frames where subsequently interpolated onto a 100-μm grid using zero padding in the frequency domain. This interpolation step helps to obtain isotropic pixels and better looking PDIs. The resulting PDI was then computed by averaging the magnitude for every complex pixel signal over all remaining frames. Having the interpolation step before computing the PDI results in a much smoother and higher-resolution PDI than when the interpolation applied on the PDI. We then checked on outlier PDIs by computing the MAD of every PDI with respect to the median over all PDIs. PDIs with a MAD score of 2 × 1.48 were replaced with a median PDI (*63*). This procedure introduced in other papers (*63*) only affected a few cases where sudden head motion gave rise to a strong outlier PDI. Subsequent subpixel motion compensation for every PDI with respect to the median PDI was applied using a cross-correlation technique that can be efficiently computed in the frequency domain (*64*). This step yielded smoother pixel signals over time and slightly higher functional correlation values. The SDs of the motion in the *x* and *z* directions for all datasets shown in Fig. 5 are 60 and 10 μm, respectively. Detailed plots of the motion for these sets are shown in data S8.

For every pixel in the PDI, the PCC “r” was computed between the stimulus signal (convolved with our estimated fUSi HRF; see above) and pixel intensity over time. A noise region was defined in each PDI, where no vascular or response signal was to be expected. Functional pixels were identified as those with PCC > 3× SD of the PCC values in the noise region. We have determined this threshold based on a heuristic approach, where we studied each dataset with different cutoffs and chose a uniform threshold we could apply across all datasets that we show in this manuscript, to ensure accurate comparison of datasets across time points. Other approaches for classifying functional pixels in fUSi have been reported in literature, such as PCC combined with a Fisher’s transform to calculate a *z* score (*65*). We produced “overlay figures,” where functional pixels were displayed over the mean PDI (grayscale).

The average hemodynamic time traces or “functional signals” that are shown in Figs. 2 to 5 depict the relative change with respect to the baseline signal, which, in our case, is defined as the mean signal amplitude over the first 10 s (*18*).

### Continuous lip brushing task

For Fig. 4K, we mapped the hemodynamic signal to the brush position with respect to the subject’s face. For this purpose, we first performed an ON-OFF test similar to what is shown in Fig. 4A to identify the functionally significant pixels. These pixels were then used in the continuous brushing test. The average hemodynamic time trace was computed as described above and was further smoothed using a 5-s moving median filter to make the mapping more robust. An average hemodynamic delay obtained from our estimated HRF was added to the tracking data time vector to ensure a hemodynamically meaningful mapping between the tracking data and ultrasound data. The hemodynamic time trace then was interpolated to the same time sampling as the tracking data. For this experiment, where we focus on the lips and a portion of the cheeks, we only used the *x* and *y* coordinates of the tracking data as the variation in depth (*z* dimension) was very minimal. These three vectors (*x* and *y* tracking coordinates and hemodynamic signal) were then used for the scatterplot shown in Fig. 4K where every *x* and *y* coordinate gets a dot which is colored by the amplitude of the hemodynamic signal. In addition, the size of every dot is scaled by the magnitude of the hemodynamic signal. The hemodynamic signal was delayed with the average hemodynamic delay (3.8 s) for the scatterplot to have an appropriate hemodynamic relation between the location on the face/lips and the expected consequential rise in the functional signal. Therefore, in Fig. 4L, the functional signal is labeled as “delayed functional signal” (*66*–*70*).
