# Supplementary material for: Mobile human brain imaging using functional ultrasound
Source: Sci Adv. 2025 Jun 18;11(25):eadu9133. doi: 10.1126/sciadv.adu9133 (PMC12175903; doi:10.1126/sciadv.adu9133)
Supplement: Supplementary file 1 — Data S1 to S10 Tables S1 to S3 Figs. S1 to S13 Legends for movies S1 and S2 [file sciadv.adu9133_sm.pdf]

Science Advances
AAAS

Supplementary Materials for

Mobile human brain imaging using functional ultrasound

Sadaf Soloukey *et al.*

Corresponding author: Pieter Kruizinga, p.kruizinga@erasmusmc.nl

Sci. Adv. **11**, eadu9133 (2025)
DOI: 10.1126/sciadv.adu9133

The PDF file includes:

Data S1 to S10
Tables S1 to S3
Figs. S1 to S13
Legends for movies S1 and S2

Other Supplementary Material for this manuscript includes the following:

Movies S1 and S2

Supplementary Data 1 – Subject Characteristics

Table S1 - Overview of the characteristics of the N=2 subjects included in this study.

Subject	Age Category	Underlying Etiology	Location SBD + PEEK	Handed-ness	Post-operative Neurological Deficits	Baseline Language Assessment	Baseline Cognitive Assessment	Number of measurements
#1	30-35 y	High-Energetic Trauma with multiple cerebral contusions left hemisphere	Hemi-craniectomy Left >4 years in situ	Right	Minimal motor deficits in right arm and leg	Severe Aphasia<br><br>Aphasia Bedside Check: 4/14 Boston Naming Test: 6/60 Token Test: 5.5/36	Complicated due to aphasia	N = 6
#2	35-40 y	Low grade Astrocytoma right insula	Fronto-temporal Right >4 years in situ	Right	None objectified or reported	No deficits	No deficits	N = 2* **Deceased during the course of the study*

A total of 2 subjects were included in this study, both males in their 30s (**Table S1**). Subject #1 received a left-sided hemicraniectomy and PEEK-implant after high-energetic trauma, causing multiple cerebral contusions and post-operative neurological deficits (**Figure S1, panel A**). Most pronounced was the subject's aphasia, which was considered severe based on the baseline linguistic assessment. Apart from minimal motor deficits in the right arm and leg, which was visible through asymmetries in his gait resulting in a right-sided limp, subject #1 had no other motor-related deficits. Subject #2 received a PEEK-implant in the right-sided frontotemporal region after surgical removal of a low grade astrocytoma in the right insular region. The tumor resection cavity was still clearly visible on MRI-scans (**Figure S1, panel B**). At time of inclusion, the subject was tumor progression-free for multiple years. No cognitive, motor or language deficits were reported or objectified at baseline. Experiments were conducted over a period of 2 years. Subject #1 participated in a total of seven measurements during this period. Subject #2 deceased during the study due to tumor regrowth and participated in two measurements.

The PEEK cranioplasty of subject #1 was custom designed to cover the left-sided hemicraniectomy. Usually, PEEK cranioplasties are modelled after the subject's contralateral, non-affected skull based on CT-scans (manufacturer Johnson&Johnson, DePuySynthes).
Overall thickness (4 mm) of the cranioplasty of Subject #1 was very consistent across the implant (**Figure S2, panel A-B**). The cranioplasty itself was 130 x 162 mm, with a 44 mm extension due to the curvature (**Figure S2, panel C-D**). What is not displayed in these models, is the final step in de cranioplasty design consisting of a Mesh-pattern of small holes (millimeter-range) to facilitate drainage and per-operative sutures (**Figure S2, panel C**). During the measurements, no particular effort was taken on our end to find an area with larger or smaller curvature. Nor did we actively avoid placing the probe on or around the holes, as we did not find it affected our PDI-quality significantly. Rather, our ROIs and probe placement were purely driven by the expected localization of functional regions of interest (sensorimotor cortices of the mouth).

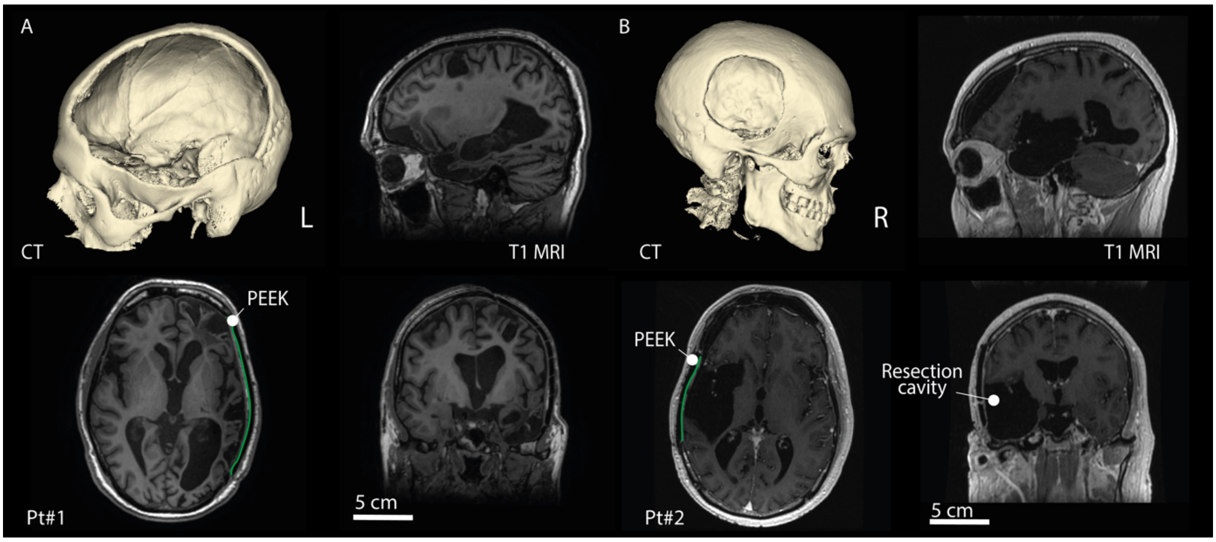

Figure S1 – CT-scans of the included subjects in this study. A) Subject #1 received a left-sided hemicraniectomy and PEEK-implant after high-energetic trauma. B) Subject #2 received a PEEK-implant in the right-sided frontotemporal region after surgical removal of a low grade astrocytoma in the right insular region.

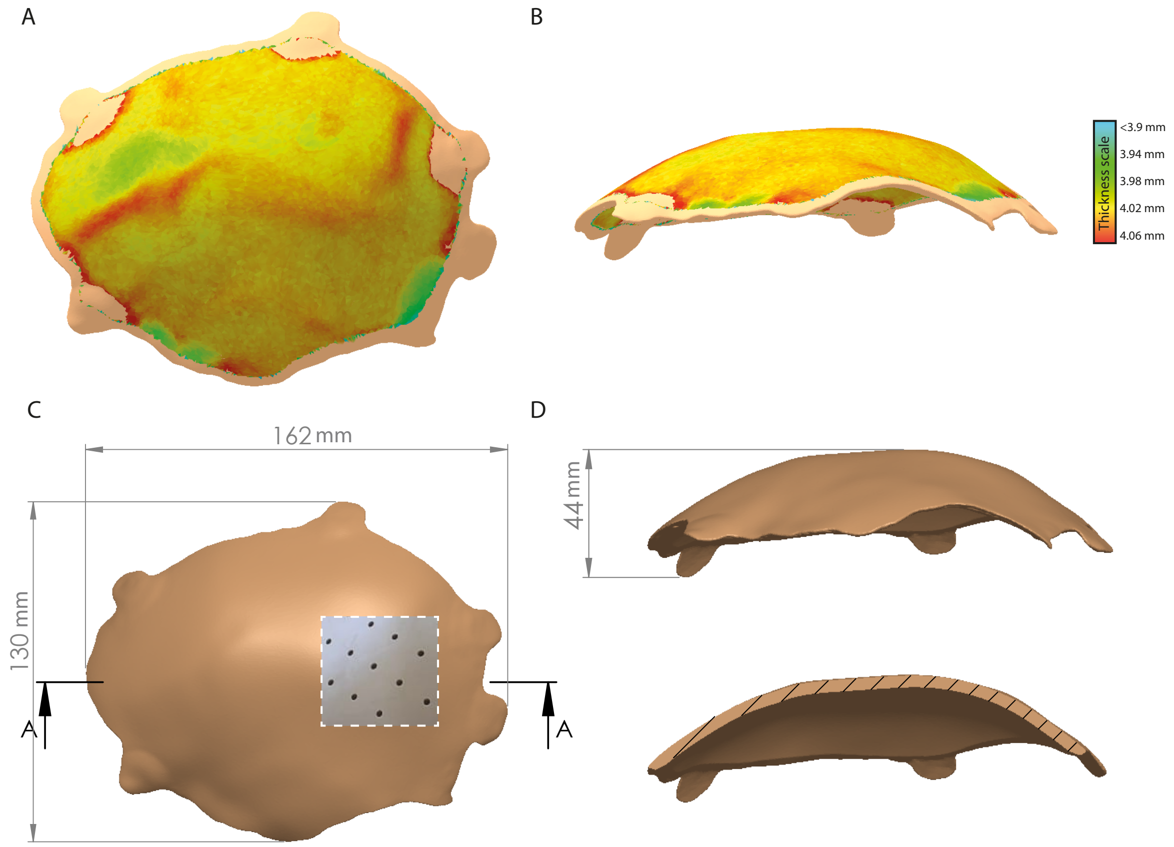

Figure S2 – PEEK cranioplasty details of subject #1. A-B) overall thickness was consistent around 4 mm. C-D) Dimensions were 130 mm x 162 mm x 44 mm. The level of curvature is displayed in an orthogonal plane. The models as displayed here do not contain the final design step of our clinical PEEKs, which consists of a consistent Mesh-pattern of small holes (1-2 mm wide), across the PEEK, which allows for drainage and suturing per-operatively.

Supplementary Data 2 – Helmet Fabrication Pipeline

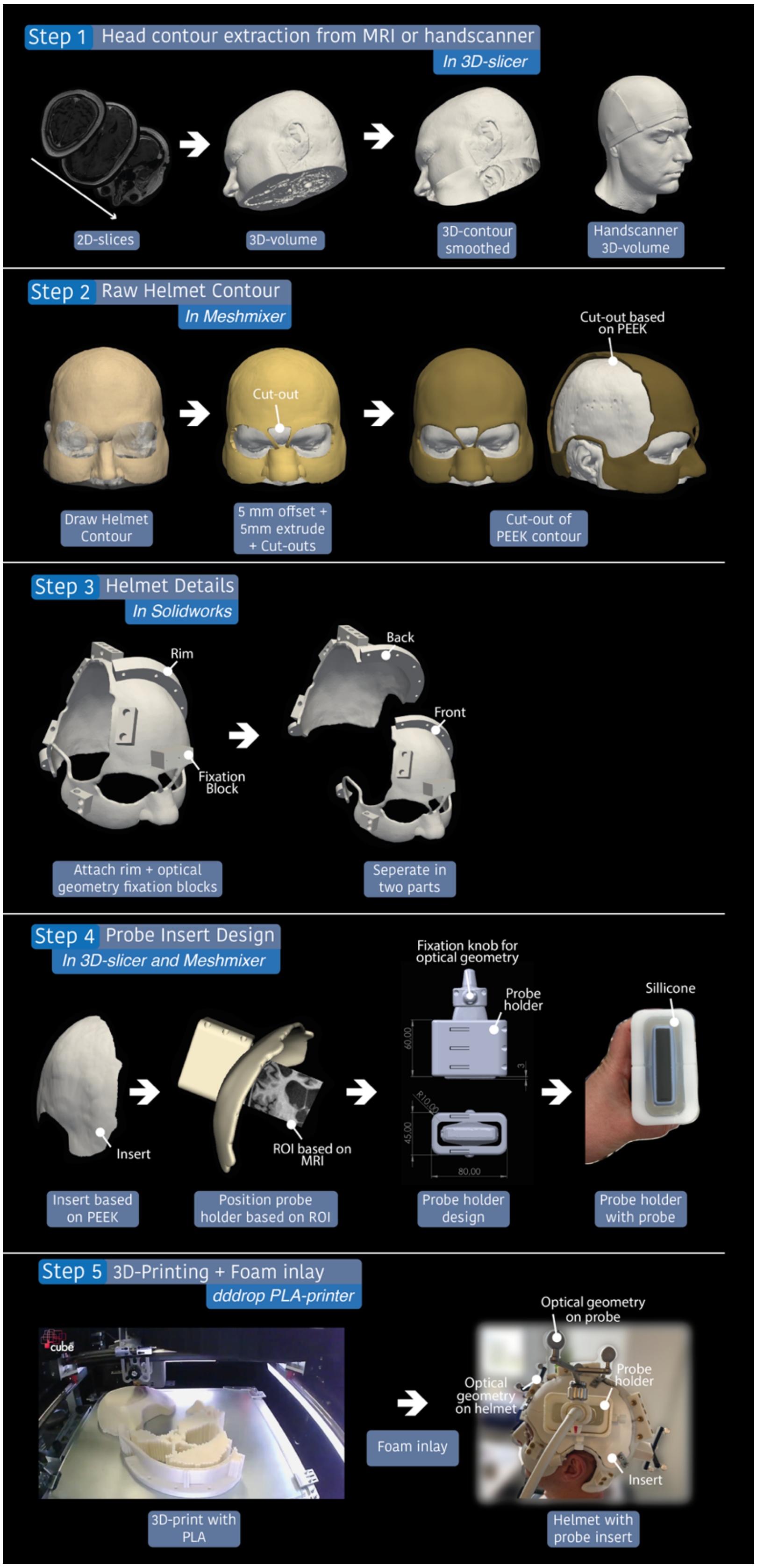

Figure S3 - Step-by-step overview of the helmet fabrication pipeline. Fabrication consisted of a total of five steps, going from head contour extraction, to online design of the helmet and probe insert, and 3D-printing.

Step 1 consisted of extracting the contour of the face and head of the subject from clinically available structural MRI datasets of each subject. If no recent MRI-scan was available, an additional 3D surface scan of the face was made using a handheld 3D-scanner and co-registered to the MRI using the landmark registration function in 3D Slicer (this was the case for subject #1).

In Step 2 the subject's head contour was smoothed in Meshmixer (Autodesk Inc.), before applying a 5mm offset and 5 mm extrusion to create the base of the fUSi-helmet. Using the same landmark registration function in 3D Slicer, the structural MRI-scan was co-registered to the subject's 3D CT-scan which was made prior to PEEK-production, in order to determine the borders of the defect. A cut-out was made in the helmet with approximately 10 mm margin around the border of the PEEK, which was used as the base for the probe insert, as will be further discussed in Step 4.

In Step 3, using SolidWorks (Dassault Systèmes, SolidWorks Corporation) the base of the helmet was expanded with a fixation rim and fixation blocks to mount the necessary geometries for optical tracking (Northern Digital Inc., Canada), see below. The fUSi-helmet was divided in two parts parallel to the fixation rim to allow for printing and fixation on the subject's head.

In Step 4, probe inserts were designed based on targeted brain regions of interests (ROIs) for functional tasks using (f)MRI or prior (f)USi-locations. The inserts ensured stability of the probe during functional tasks as well as enable reproducibility of the same 2D-imaging plane across measurements. In order to ensure proper fixation of the ultrasound probe inside this insert, a rectangular probe holder with silicone-lining was molded to the shape of the probe, allowing for fixation of the probe inside the insert.

In Step 5, the helmet was 3D-printed in-house using PLA-material (dddrop bv, CAD2M). Finally, the inside of the 3D-printed helmet was covered in foam to ensure subject comfort. Before probe holder containing the probe was positioned in the insert, care was taken to cover the exposed portion of the skull below the insert in a layer of warmed ultrasound-gel, to facilitate optimal acoustic contact.

Supplementary Data 3 – Optical Tracking Pipeline

Position and orientation of the fUSi-probe and helmet was tracked continuously using an NDI Polaris Vega optical tracking system (SN P9-04539, Northern Digital Inc., Canada), which was configured to track infra-red reflective reference geometries attached to the fUSi-probe and helmet. Custom software was designed to record the tracking information featuring six degrees of freedom (DOF) at an average rate of 20 Hz.

The computation of reference (f)MRI scan slices that corresponded to the ultrasound images required a sequence of transformations on the tracking data. These transformations, provided by the tracking system and through calibration of the tools, facilitated the mapping of a 2D pixel coordinate *(i, j)* in an ultrasound image at a distinct time point *k* to a 3D voxel coordinate *(u, v, w)* in the reference image. The transformation series is described as:

$$\begin{bmatrix} u \\ v \\ w \\ 1 \end{bmatrix} = \mathbf{T}_{\text{img}}^{-1} \cdot \mathbf{T}_{\text{reg}} \cdot \mathbf{T}_{\text{ref},k}^{-1} \cdot \mathbf{T}_{\text{us},k} \cdot \mathbf{T}_{\text{us,local}} \cdot \mathbf{T}_{\text{us,img}} \cdot \begin{bmatrix} i \\ 0 \\ j \\ 1 \end{bmatrix} \quad (S1)$$

In this formulation, each **T** embodies a 4x4 affine transformation matrix, encapsulating rotation and translation parameters. Each transformation matrix's role in the sequence is explicated as follows, moving from right to left:

$$\mathbf{T} = \begin{bmatrix} r_{11} & r_{12} & r_{13} & t_x \\ r_{21} & r_{22} & r_{23} & t_y \\ r_{31} & r_{32} & r_{33} & t_z \\ 0 & 0 & 0 & 1 \end{bmatrix} \quad (S2)$$

1. $\mathbf{T}_{\text{us,img}}$: Alters pixel coordinates in the ultrasound image into physical positions by scaling them relative to the pixel dimensions and centering the image at the upper middle of the frame.

2. $\mathbf{T}_{\text{us,local}}$: Adjusts the position on the reference geometry attached to the ultrasound probe to align with the center of the ultrasound array.

3. $\mathbf{T}_{\text{us},k}$: Indicates the measured position and orientation of the reference geometry on the fUSi-probe in relation to the tracking camera at time point *k.*

4. $\mathbf{T}_{\text{ref},k}$: Specifies the measured position and orientation of the reference geometry on the fUSi-helmet relative to the tracking camera at time point *k*.

5. $\mathbf{T}_{\text{reg}}$: Denotes the registration transformation mapping the position of the reference geometry on the fUSi-helmet to its position within the anatomical coordinates of the reference (f)MRI image.

6. $\mathbf{T}_{\text{img}}$: Transforms voxel coordinates in the reference image into anatomical coordinates.

To assist in locating specific anatomical regions during hand-held ultrasound scans, we utilized the tracking information in conjunction with software from the Visualization Toolkit (VTK) (*61)*. This combination allowed real-time visualization of the fUSi-probe's position concerning the helmet and several anatomical ROIs, as well as the MRI slice that corresponds to the current ultrasound image (see **Supplementary Data 4**). The tracking data was also saved for later use, facilitating offline 3D reconstruction of the ultrasound path, as demonstrated in **Figure 1B**.

Supplementary Data 4 – Parallel Datastreams recorded

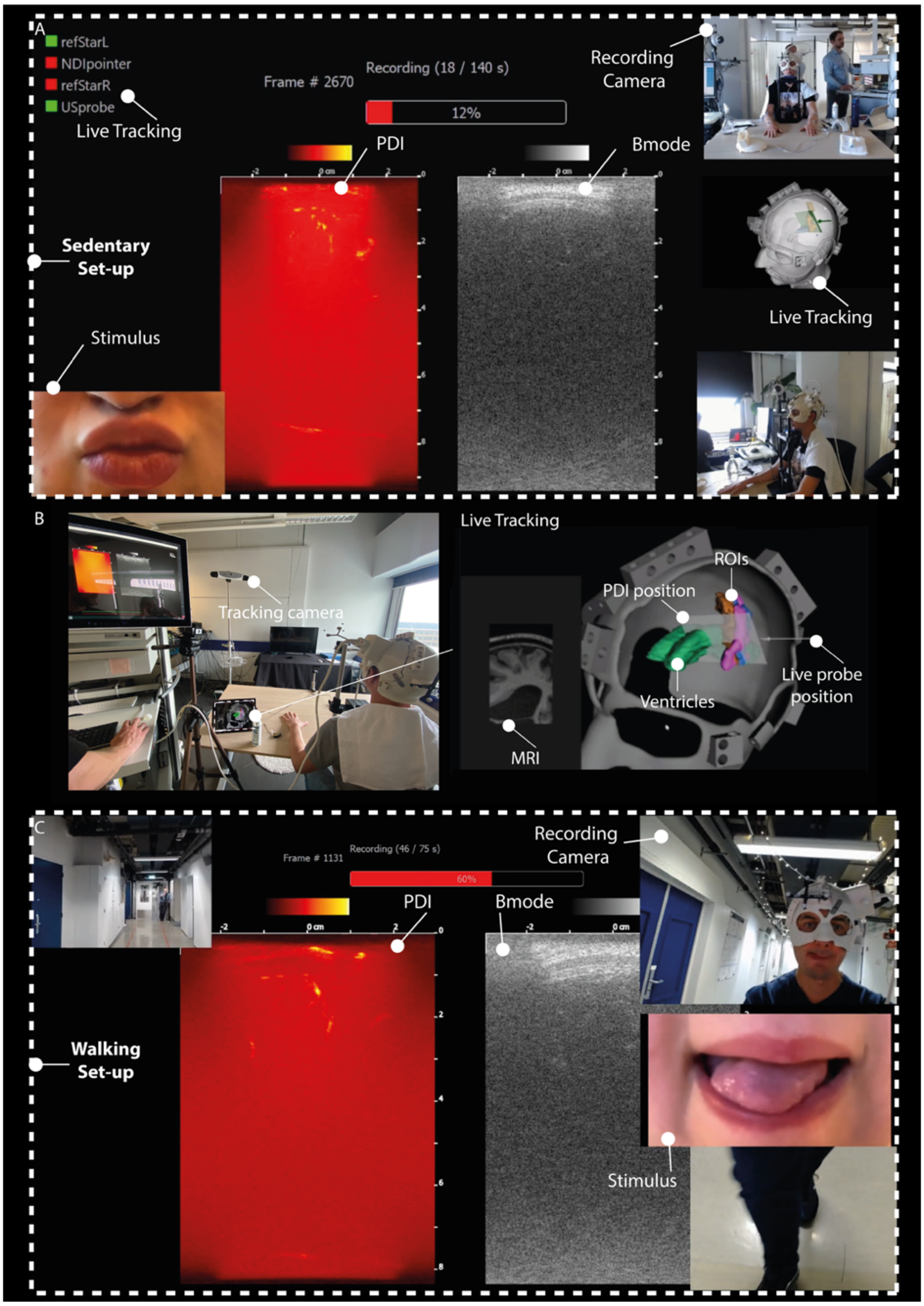

Figure S4 – Overview of the parallel data-streams acquired and stored during our experiments. The upper panel concerns our sedentary experiments in the lab, while the bottom panel concerns the measuremens during locomotion.

The live PDI and Bmode-image as acquired with our custom acquisition unit were recorded in parallel with optical tracking data of the position of the ultrasound probe relative to the patient's helmet and brain anatomy. A live tracking tool was made using VTK (*61*) to display the probe position relative to the patient anatomy in real-time using previous MRI-scans of the patient (middle panel). Multiple camara-streams were recorded in parallel, as well as the real-time functional stimulus video shown to the patient. This allowed us to study the patient's task performance offline and adjust the stimulus pattern or stimulus delays accordingly.

Supplementary Data 5 – Ultrasound Distortion Correction

In this study it was possible to visualize functional activity through the PEEK-cranioplasty. However, while the homogeneity of the implant permits imaging compared to an intact skull it still possesses drawbacks compared to soft-tissue imaging. The interfacial losses caused by transmission through the inset and its higher bulk attenuation both act to decrease the SNR thereby reducing the imaging depth. The impact of these losses on imaging through PMMA has been investigated in a previous study (Rabut et al, 2024) (*33*). In addition to these interfacial losses, however, the higher bulk sound speed and complex shape of the implant also induces a lensing effect on the transmitted wave-field. This can be seen in (**Figure S5**) below which shows a measurement of the acoustic field generated when transmitting a planewave on the clinical transducer both with and without the skull implant. These measurements were taken using a 200 µm needle hydrophone in a custom-built scanning tank. In comparison to free space the shape of the transmitted wavefront is warped, and the time-of-arrival is also shifted.

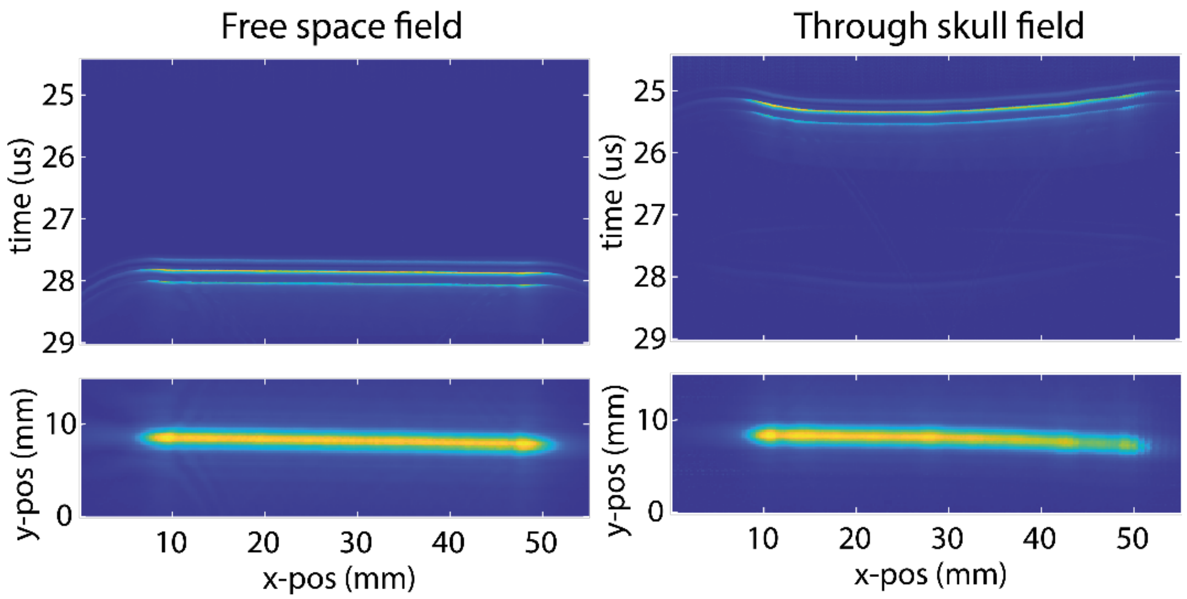

Figure S5 – **Measurement of the acoustic field generated when transmitting a planewave on the clinical transducer both with and without the skull.** (left) Hydrophone scan of acoustic field generated by GE9LD transmitting a normal plane wave in free space. (right) Hydrophone scan of acoustic field generated by GE9LD transmitting a normal plane wave after propagation through skull inset

This distortion of the wavefront and change in time-of-arrival both affect the image reconstruction. First, it makes it necessary to increase the bulk sound speed used in the delay and sum algorithm to compensate for the higher sound speed of the skull implant (2750 ms^{-1} vs 1550 ms^{-1}). This increase in the speed of sound can refocus the image, however, only over a limited region as the shift required is proportional to the fraction of the wave path occupied by the skull so changes with depth. Second, by ignoring the lensing of the transmitted wavefront the position and shape of the reconstructed vasculature will be warped compared to its true position. Both effects are illustrated by **Figure S6** which shows reconstructed images of pulse-echo data simulated from a grid of points positioned below a skull implant. Pulse-echo data from the grid of points (**Figure S6, panel A**) was simulated using the k-Wave toolbox *(66)*, which uses a k-space pseudo-spectral model for time domain simulations of acoustic waves. Images were reconstructed using a delay-and-sum approach using two different values

of bulk sound speed - 1700 ms^{-1} (**Figure S6, panel B**) and 1600 ms^{-1} (**Figure S6, panel C**). As the sound speed decreases the depth at which the points are in-focus increases, as the skull occupies a lower-fraction of the wave path. The overall position of the points is also tilted compared to their true position due to the lensing effect of the skull.

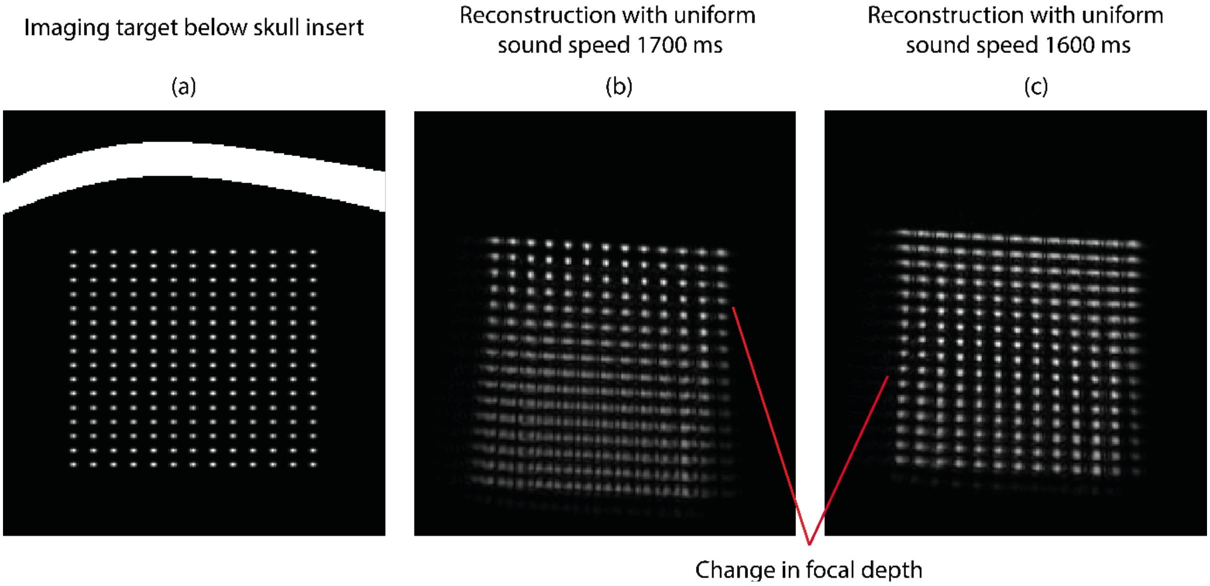

Figure S6 - Lensing effect of skull insert on reconstructed image. Lensing effect of skull insert on reconstructed image. A) Grid of points behind skull-insert from which pulse-echo data was simulated using the k-Wave toolbox *(66).* B-C) Increased sound speed of the skull insert can be compensated by increasing the sound speed, however, this results in an image that is only partially in focus. The underlying image structure is also warped.

We investigated whether it was possible to compensate for this by using a model-based approach for image reconstruction. For this model-based reconstruction approach we assume our data y can be linearly related to our image x via a matrix vector multiplication $y = Ax$, where the matrix A contains the pulse-echo impulse response for each pixel in the imaging medium that describes the propagation of waves from the ultrasound transducer into the medium then back to the transducer.

This framing has been previously applied to conventional soft-tissue ultrasound imaging and, for example, permits regularization of the image reconstruction with the choice of an appropriate prior (67, 68). For soft-tissue imaging it can be assumed that the medium is homogeneous and each element is identical which allows for the system matrix to be computed geometrically. For this work, however, in our imaging system we have a skull implant made from a hard plastic (PEEK) with a significantly higher speed of sound, so this assumption is no longer valid and we need to incorporate the changes into the model A.

We then tried to approximate a matrix A that incorporated the effect of the implant using an image-guided approach. First, we reconstructed an image using a model A calculated assuming a homogeneous sound speed of 1550 ms^{-1} matching soft tissue. (**Figure S7, panel A**). To calculate A we simulated the forward field for each element of the linear array using k-Wave *(66)* then constructed the pulse-echo impulse response assuming linearity and reciprocity using an approach reported previously shown by Brown et al. *(62)* in a paper on computational ultrasound. Briefly, we assume that the propagation is linear - so the transmitted field for any

arbitrary transmission scheme can be computed via a simple delay and summation of the individual element fields, and each element follows reciprocity - so the pulse-echo response can be computed via a temporal convolution of the transmitted field with the individual element responses.

The image was reconstructed using matched filter (e.g., $x = A^H y$). This generated an image that was largely out of focus (**Figure S7, panel B**), however, could be used to manually segment the top-surface of the skull implant. Using this segmented surface we generated a new sound speed map for our simulation domain comprising a half-space of soft-tissue and the skull insert (**Figure S7, panel C**). The model was then recomputed in this half-space using k-Wave *(66)* to model the wave propagation and a second B-mode image was reconstructed from which the bottom surface of the skull implant could be manually segmented (**Figure S7, panel D**). With the segmented bottom surface, we were able to generate a final map of acoustic properties containing the full skull-bone implant shape. The model was again updated using k-Wave *(66).* This step-by-step procedure leads to a model matrix A in which the acoustic propagation of sound waves from the transceivers through the skin, lens and brain and back again are properly modelled. This procedure is not per se new as it stands in a long tradition of ultrasound imaging and focusing through heterogeneous acoustic speed maps *(57, 69, 70).*

We found that when using this final model the resulting B-mode image was visually sharper throughout the field-of-view having compensated for the lensing of the skull insert (**Figure S7, panel F**).

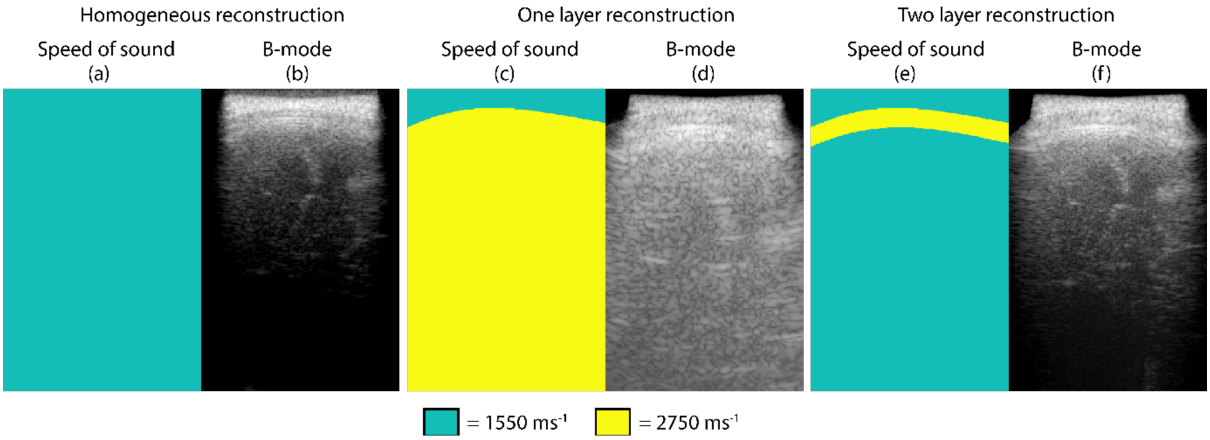

Figure S7 - Image-guided approach used to correct for the lensing effect of the skull insert. A) sound speed map and B) resulting B-mode image used for segmenting the first skull layer. C-D) sound speed map and resulting B-mode image used for segmenting the second skull layer. E-F) final sound speed map and B-mode image that corrects for the lensing effect of the skull insert.

We recomputed one set of PDIs using the updated model using 200 frames of raw RF data. For comparison we also reconstructed a PDI from the same data using a bulk sound speed of 1700 ms^{-1}. It can be seen in **Figure S8** that the resulting visual correspondence is significantly improved.

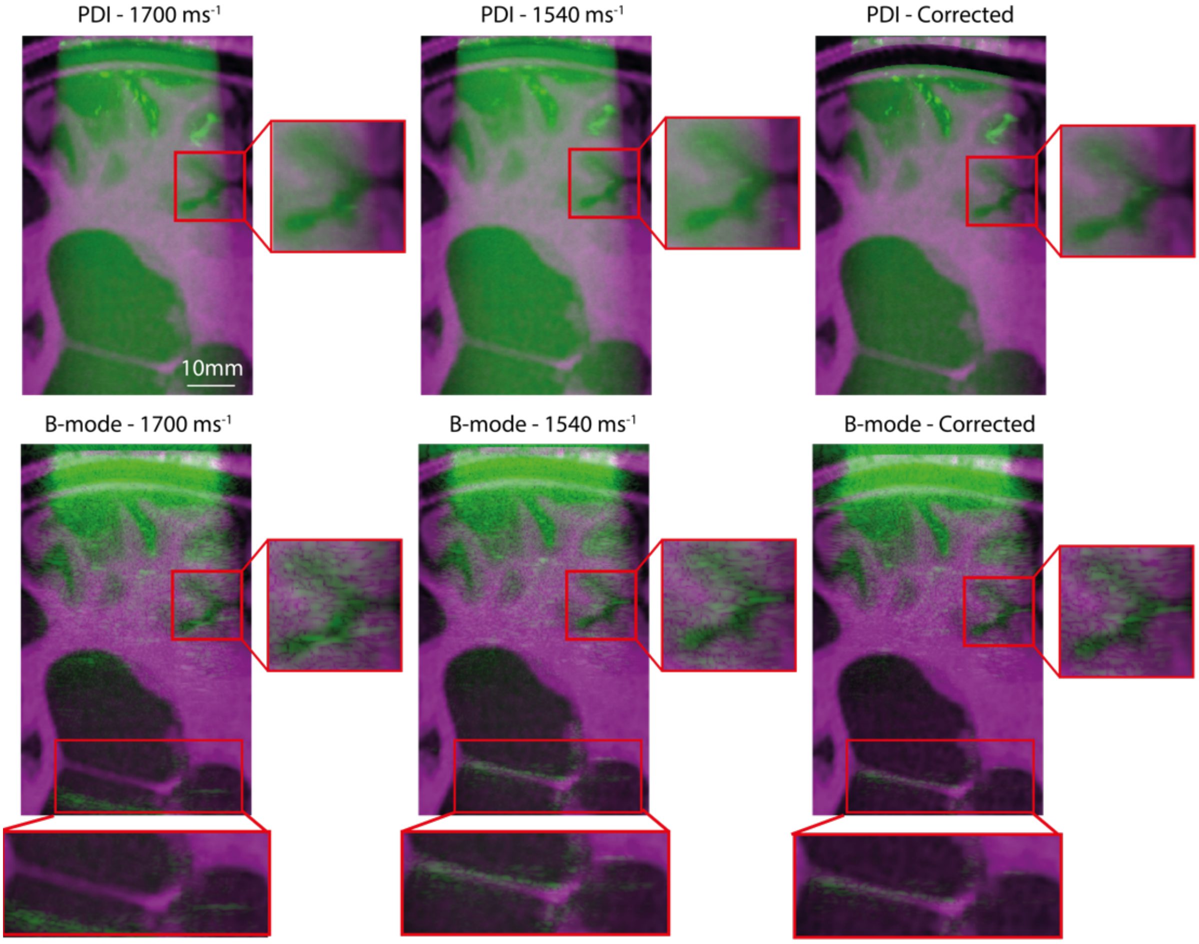

Figure S8 – Comparing the results of our corrected PDI, versus bulk sound speed reconstructions. Results of our improved visual correspondence between the PDI corrected using our proposed methods (most right panel), versus the PDI reconstructed using a bulk sound speed of 1700 ms^{-1} or 1540 ms^{-1} (left and middle panel, respectively).

Supplementary Data 6 – Hemodynamic response function estimation

The temporal fluctuations of the functional signal *y(t)* recorded during tasks is commonly modelled as the response of a linear time-invariant (system) to the task time course *u(t).* The impulse response of the LTI system is known as the hemodynamic response function (HRF) $h^{(1)}(t)$. Considering a certain baseline fUSi signal $h^{(0)}$, the functional signal time series can be written as:

$$y(t) \approx h^{(0)} + \sum_{t_1=0}^{T-1} h^{(1)}(t_1)u(t-t_1) \;\; (S3)$$

Based on the above mathematical model, and using the fUSi measurements as well as the known stimulus time course, the parameters h(0) and h(1)(t) can be estimated. To ensure that the estimated parameters produce a plausible HRF shape, the kernel h(1)(t) can be expanded as the linear combination of L temporal basis functions b_i(k), k=1,..,L. The basis functions were chosen in the form of gamma functions:

$$b(k;\boldsymbol{\theta}) = \theta_1\left(\Gamma(\theta_2)\right)^{-1}{\theta_3}^{\theta_2}k^{\theta_2-1}e^{-\theta_3 k} \;\; (S4)$$

The parameters were varied to account for different possible peak delays, equidistant from the stimulus onset. The basis functions for L=8 are depicted in **Figure S9**. The detailed estimation methodology is described in Kotti et al. (2023) (*43),* with the difference that in this work the kernel was not restricted to be positive only.

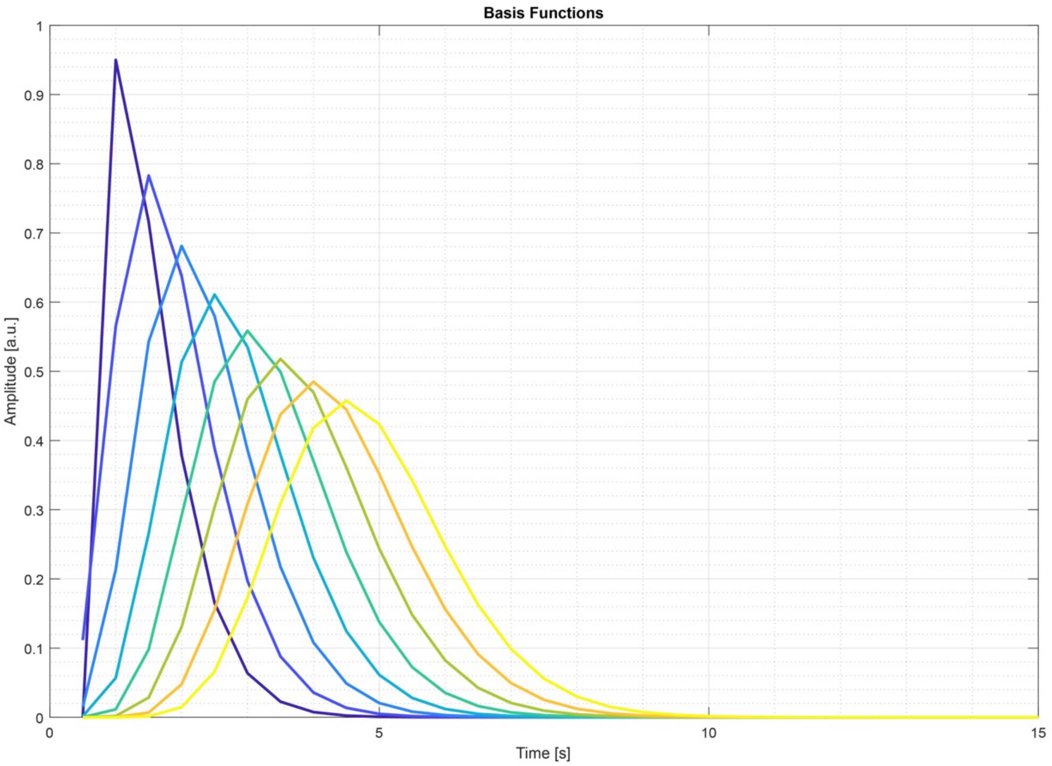

Figure S9 - **Basis functions with L = 8 (*see S3 and S4*).**

We used a total of N = 4, 55s long fUSi recordings for HRF estimation. The optimal number of basis functions L, as well as the l1-regularization coefficient λ controlling the number of basis functions with non-zero weight, were determined in a 4-fold cross-validation setting. Namely, we concatenated all but one measurement for estimating the HRF, then convolved the tracking time course with the estimated HRF to predict the left-out fUSi time series using equation (1). To evaluate the quality of the estimation, the PCC and mean squared error between the predicted and measured fUSi were calculated. Based on this cross-validation, the values L=10 and λ=0.1 were chosen. After establishing these optimal values, all 4 measurements were concatenated to estimate the final HRF to be used in our further analysis. The resulting HRF is shown in **Figure S10** (dotted green line) overlayed on estimated HRFs from each fold in the cross-validation (thin lines).

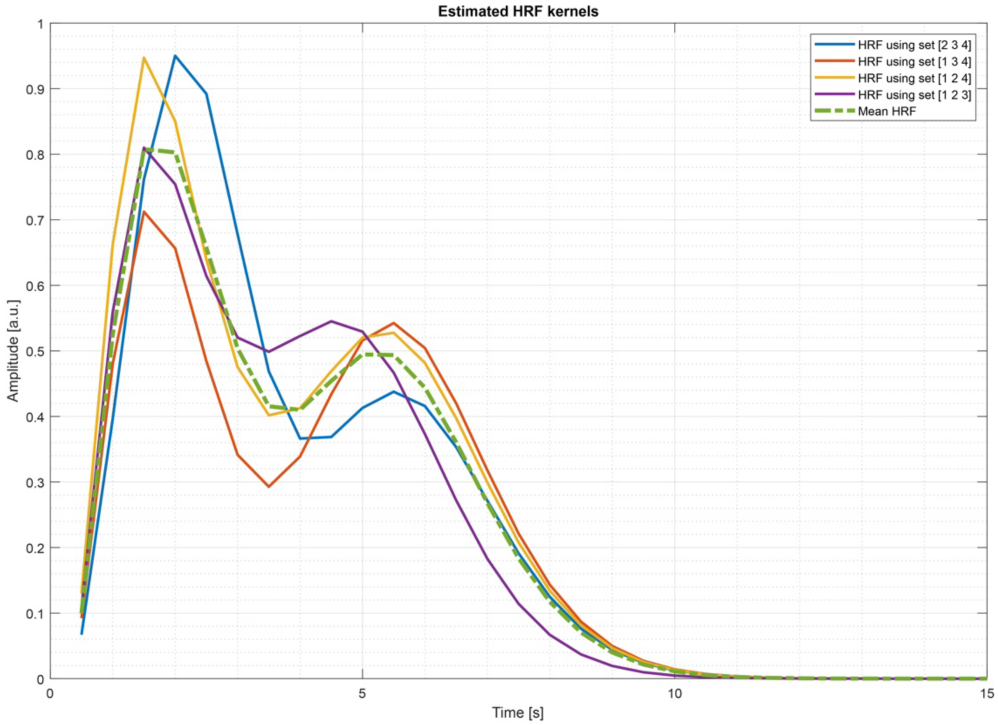

Figure S10 - Estimated HRF based on all measurements (dotted green) overlayed on estimated HRF from each fold in the cross-validation (thin lines).

To illustrate the reliability of the estimated fUSi time courses using the above HRF, we applied this HRF in an independent set of measurements. In **Figure S11** below, the raw fUSi time courses are shown in blue, the tracking time course (shifted with 3.5s) in black and the estimated fUSi time course in orange. The estimated fUSi time series achieves higher correlations with the measured time course compared to the optimally delayed tracking signal (r_hrf and r_raw, respectively, shown above the time traces).

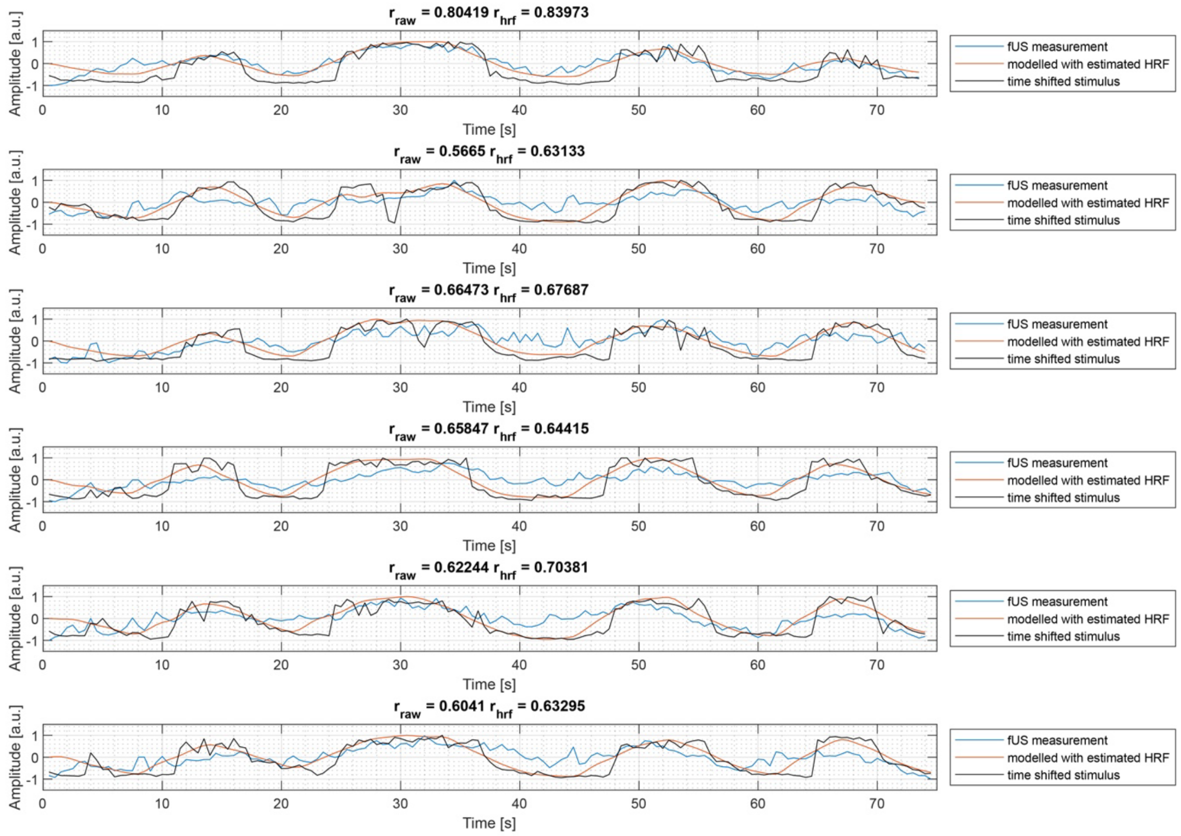

Figure S11 - Overview of the estimated fUSi time courses using the estimated HRF shown in Figure S10. The estimated fUSi time courses aare shown in orange. For comparison, measured fUSi time courses are shown in blue and the tracking time course (delayed with an optimal delay of 3.5s) is shown in black. Correlations between the fUSi measurements and the estimated fUSi time courses (r_hrf) are higher than correlations between the measurements and the optimally delayed tracking time courses (r_raw).

Supplementary Data 7 – Overview of functional tasks used during fUSi and fMRI

Table S2 – Details of each of the functional tasks used during fUSi and fMRI.

Figure Panel	Modality	Task Content	Task Pattern	ON-task	OFF-task
2E	fMRI	Lip Pouting (motor, video-guided)	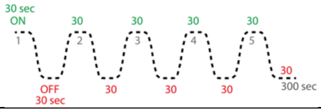	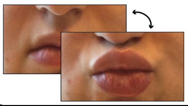	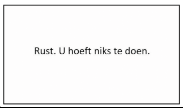
2D	fUSi	Lip Pouting (motor, video-guided)	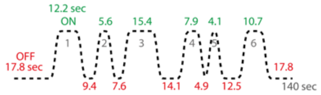	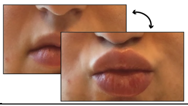	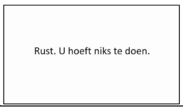
2H	fUSi	Lip Sensory (brushing by experimenter)	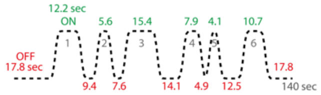	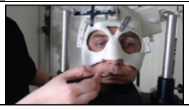	
3C	fUSi	Lip Pouting (motor, video-guided)	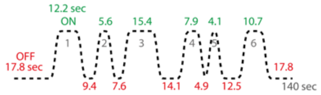	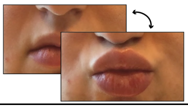	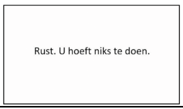
4A	fUSi	Lip Sensory (brushing by experimenter)	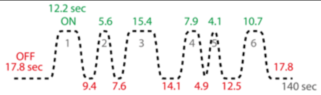	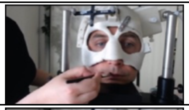	
4B	fUSi	Lip Sensory Forehead in off (brushing by experimenter)	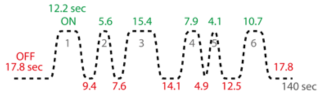	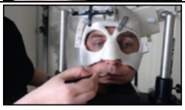	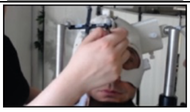
4C	fUSi	Lip Sensory Ear in off (brushing by experimenter)	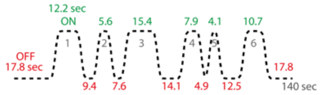	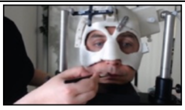	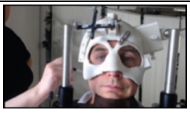
4D	fUSi	Lip Sensory Hand in off (brushing by experimenter)	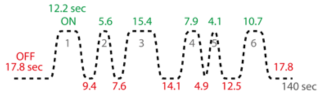	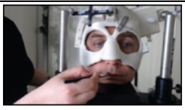	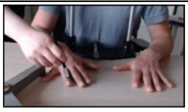
4E	fUSi	Forehead Sensory (brushing by experimenter)	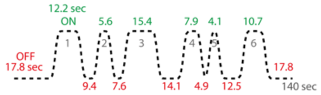	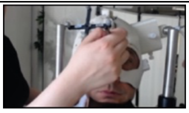	
4F	fUSi	Lip Pouting (motor, video-guided)	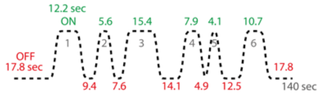	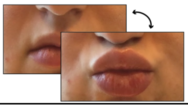	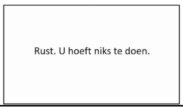
4G	fUSi	Lip Sensory Imagined (Video-guided)	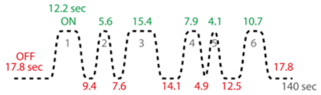	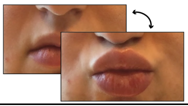	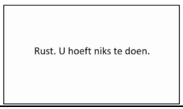
4K	fUSi	Lip Sensory (continuous brushing by experimenter)	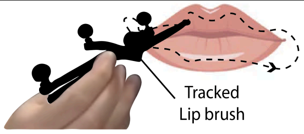	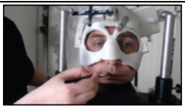 *Continuous*	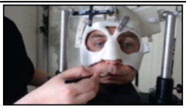 *Continuous*
5D	fUSi	Lip Licking (video-guided)	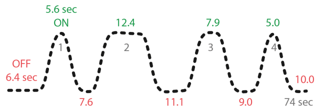	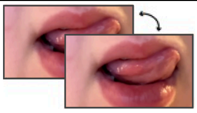	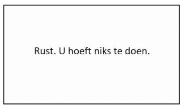
5F	fUSi	Lip Licking (video-guided)	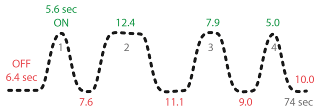	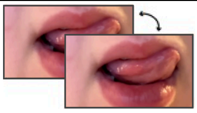	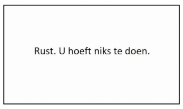

5G	fUSi	Lip Licking (audio-guided)	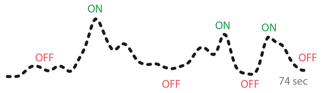<br>*Depending on the tracked lip trace*	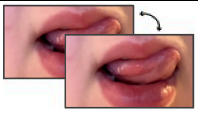	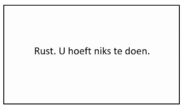
5H	fUSi	Lip Licking (imagined)	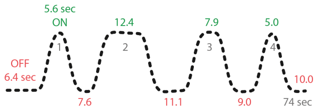	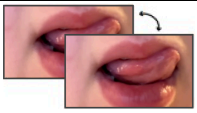	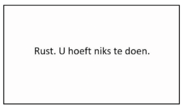
5I	fUSi	Finger Tapping (imagined)	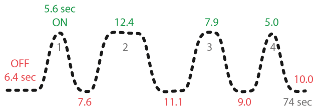	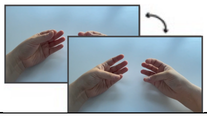	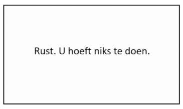
5J	fUSi	Data from 5G, 5H and 5I combined in one scatterplot	*See above*	*See above*	*See above*

Supplementary Data 8 – Average motion during walking tasks

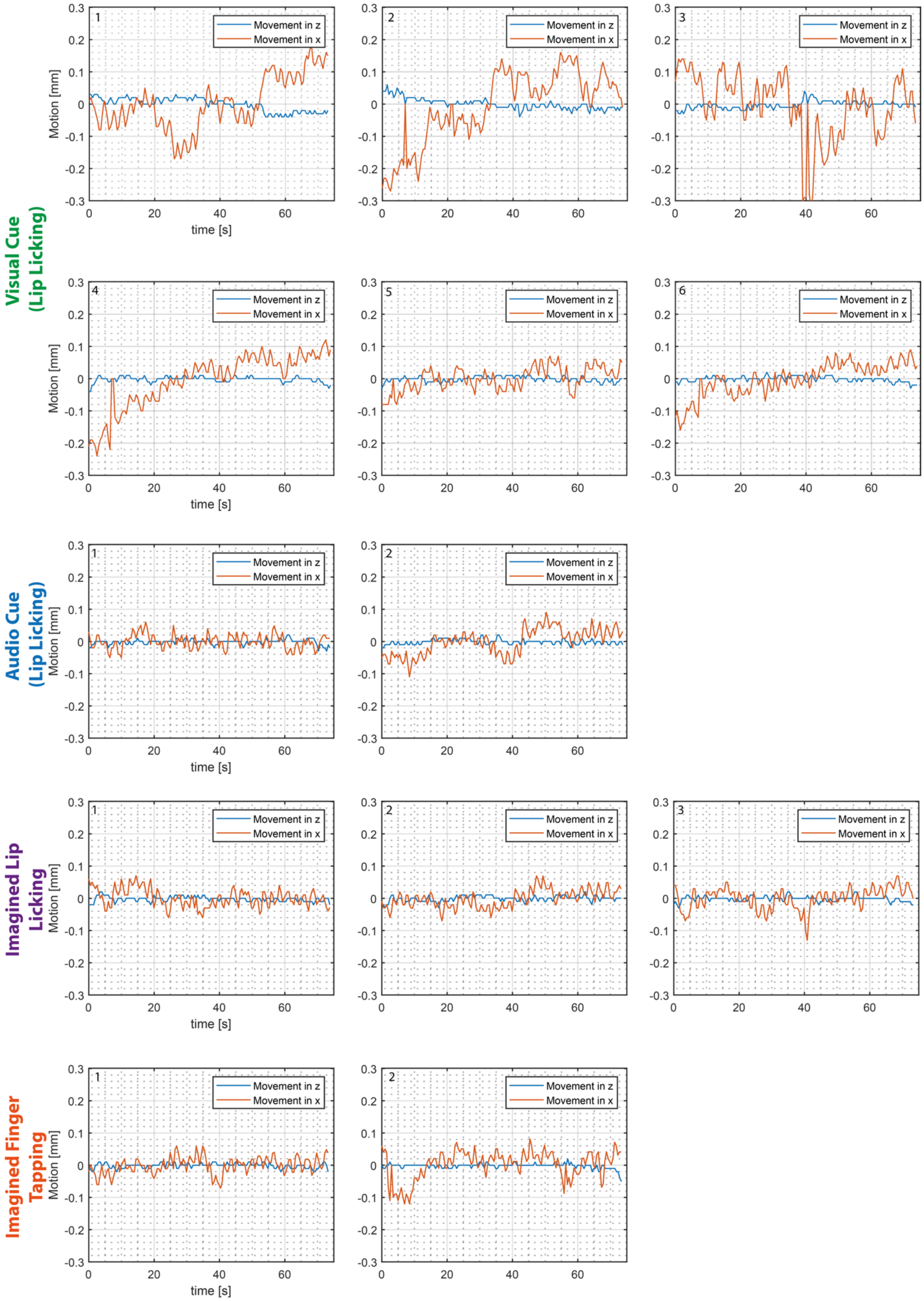

Figure S12 – Overview of the average in-plane movement (in z- and x-direction, based on the PDIs) during each of the walking task variations. Any movement is consistently in the submillimeter-range, indicating stable fixation of the probe over our 2D-ROI using the helmet.

Supplementary Data 9 – Conventional ultrasound images through PEEK

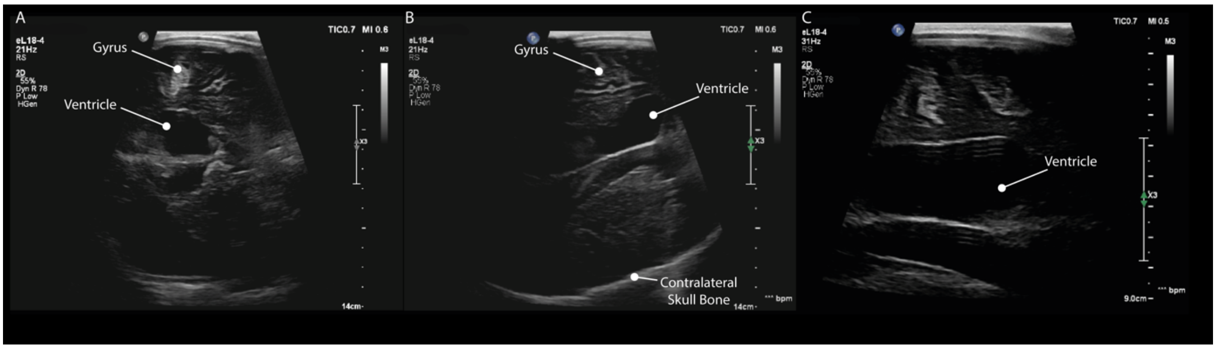

Figure S13 – **Overview of B-mode images that could be acquired through PEEK using a commercially available ultrasound machine**.

In order to put our fUS-images in perspective, we made images through PEEK in pt.#1 using a commercial-grade ultrasound machine (Philips EPIQ Elite), interfaced with an eL18-4 linear array (Philips, 1920 elements, 21 MHz center frequency). The sonotransparancy of the PEEK is confirmed again with the Bmode images we were able to make, showing even the contralateral skull bone in view (see **Figure S13**).

Supplementary Data 10 – Datasheet

Table S3 – Details of each of the datasets displayed in this manuscript.

Figure Panel	Subject	Content	Recording ID (internal code)	Date of data collection	Recording Duration
1C	#1	Free-hand sweep over cranioplasty (vascular data only)	522	10-05-2023	49 sec
2B	#1	Motor lip pouting task (ON (lip pouting) – OFF (nothing))	546<br>**Ne = 600*	10-05-2023	140 sec
2C	#1	Motor lip pouting task (ON (lip pouting) – OFF (nothing))	444 | 546| 20240902T151431<br>Ne = 800	07-04-2022| 10-05-2023| 02-09-2024	140 sec
2D	#1	Motor lip pouting task (ON (lip pouting) – OFF (nothing))	444 | 546| 20240902T151431	07-04-2022| 10-05-2023| 02-09-2024	140 sec
2G	#1	Sensory lip brushing task (ON (lip brushing) – OFF (nothing))	531|551	10-05-2023| 10-05-2023	140 sec
2H	#1	Sensory lip brushing task (ON (lip brushing) – OFF (nothing))	531|551	10-05-2023| 10-05-2023	140 sec
3B	#2	Motor lip pouting task (ON (lip pouting) – OFF (nothing))	467	13-04-2022	140 sec
3C	#2	Motor lip pouting task (ON (lip pouting) – OFF (nothing))	467|507	13-04-2022 | 06-07-2022	140 sec
4A	#1	Sensory lip brushing task (ON (lip brushing) – OFF (nothing))	531	10-05-2023	140 sec
4B	#1	Sensory lip brushing task (ON (lip brushing) – OFF (forehead))	537	10-05-2023	140 sec
4C	#1	Sensory lip brushing task (ON (lip brushing) – OFF (ear))	544	10-05-2023	140 sec
4D	#1	Sensory lip brushing task (ON (lip brushing) – OFF (hand))	533	10-05-2023	140 sec
4E	#1	Sensory lip brushing task (ON (forehead) – OFF (nothing))	540	10-05-2023	140 sec
4F	#1	Motor lip pouting task (ON (lip pouting) – OFF (nothing))	546	10-05-2023	140 sec
4G	#1	Imagined Lip Licking (look at video cue with ON-OFF lip licking without motor movement)	529	10-05-2023	140 sec
4K	#1	Lip Sensory<br>(continuous brushing by experimenter)	621	13-12-2023	178 sec
4L	#1	Lip Sensory<br>(continuous brushing by experimenter)	621	13-12-2023	178 sec
5C	#1	Lip Licking while walking (video-guided, ON (lip licking), OFF (nothing))	640	20-12-2023	75 sec
5D	#1	Lip Licking while walking (video-guided, ON (lip licking), OFF (nothing))	640	20-12-2023	75 sec
5E	#1	Lip Licking while walking (video-guided, ON (lip licking), OFF (nothing))	639 | 640 | 641| 642 | 643 | 644	20-12-2023	75 sec
5F	#1	Lip Licking while walking (video-guided, ON (lip licking), OFF (nothing))	639 | 640 | 641| 642 | 643 | 644	20-12-2023	75 sec
5G	#1	Lip Licking while walking (audio-guided, ON (lip licking), OFF (nothing))	645 | 646	20-12-2023	75 sec
5H	#1	Imagined Lip Licking while walking (look at video cue with	647 | 648 | 649	20-12-2023	75 sec

		ON-OFF lip licking without motor movement)			
5I	#1	Imagined finger tapping while walking (look at video cue with ON-OFF finger tapping without motor movement)	650 | 651	20-12-2023	75 sec
5J	#1	*See above*	All the datasets of panels 5G-I combined	*See above*	*See above*
Suppl. Data 5	#1	Motor lip pouting task (ON (lip pouting) – OFF (nothing))	546	10-05-2023	140 sec
Suppl. Data 6	#1	Lip Licking while walking (video-guided, ON (lip licking), OFF (nothing)) (prior dataset recorded for training purposes)	607 |608 | 609 | 610<br><br>639 | 640 | 641| 642 | 643 | 644	13-12-2023<br><br>20-12-2023	56 sec<br><br>75 sec
Suppl. Data 8	#1	*See above*	All the datasets of panels 5G-I combined	*See above*	*See above*

**Ne = Ensemble Size. If no Ne is defined, the standard Ne = 800 was applied.*

Supplementary Video 1 – Showcase of PDIs acquired during a linear ultrasound path. The ultrasound probe was moved manually over a region of interest of the PEEK of subject #1. Using optical tracking, we could reconstruct the position of the probe over time, relative to the patient's anatomy. In this video, we have reconstructed the 2D-PDIs at each of the probe locations during this manual sweep.

Supplementary Video 2 – Example recording of lip licking during walking. During this task, subject #1 was asked to perform an ON-OFF lip licking task based on a visual cue, as also shown in the left small panel. Using the MediaPipe library by Google, we could track the subject's lip movements (white dotted trace). The expected hemodynamic response (tracked lip movement signal convolved with the HRF) is shown in red. The functional signal, defined as the average signal of all the functional pixels (>3x std of the noise signal) in the ROI, is displayed in green. The functional activation map corresponding to this lip licking task, updated as the number of functional datapoints increases, is shown the right small panel.
